# Temporal transcriptome profiling of developing seeds reveals candidate genes involved in oil accumulation in safflower (*Carthamus tinctorius* L.)

**DOI:** 10.1186/s12870-021-02964-0

**Published:** 2021-04-15

**Authors:** Dandan Li, Qing Wang, Xin Xu, Jingsheng Yu, Zhiyu Chen, Bo Wei, Wei Wu

**Affiliations:** 1 Agronomy College, Sichuan Agricultural University, Wenjiang, 611130 Chengdu, Sichuan People’s Republic of China; 2 Agronomy College, Guizhou University, Huaxi, 550025 Guiyang, Guizhou People’s Republic of China

**Keywords:** Transcriptome, Developing safflower seeds, Molecular mechanisms, Safflower

## Abstract

**Background:**

The investigation of molecular mechanisms involved in lipid metabolism plays a critical role for the genetic engineering of safflower (*Carthamus tinctorius* L.) to increase the oil accumulation level or to change the oil composition. Although transcript sequences are currently available for the leaves and flowers of safflower, a wide range scan of temporal transcripts at different stages of seed development has not been conducted for safflower.

**Results:**

In this study, temporal transcriptome sequencing was executed at 10, 14, 18, and 22 days after flowering (DAF) to uncover the molecular networks concerned in the biosynthesis of unsaturated fatty acids (USFAs). The results revealed that the biosynthesis of fatty acids is a dominant cellular process from 10 to 14 DAF, while degradation mainly happens after 18 DAF. Significant expression changes of two genes, stearoyl-[acyl-carrier-protein] 9-desaturase gene (*SAD*) from 10 to 14 DAF and oleate desaturase (*FAD2–1*) from 14 to 18 DAF, were detected at the transcriptomic levels, and the temporal expression patterns revealed by the transcriptomic analysis were confirmed using quantitative real-time PCR experiments. In addition, 13 candidate transcription factors (TFs) involved in regulating the expression level of the *FAD2–1* gene were identified.

**Conclusions:**

These results create a link between fatty acid biosynthesis and gene expression at different developmental stages of the seeds, provide insight into the underlying lipid metabolism, and meanwhile lay an important foundation for the genetic engineering of safflower varieties. We have identified novel candidate genes, including TFs, that are worthy of further exploration.

**Supplementary Information:**

The online version contains supplementary material available at 10.1186/s12870-021-02964-0.

## Background

Safflower (*Carthamus tinctorius* L.) is a multipurpose plant with excellent medicinal and oil value. It is well known that safflower is used in traditional Chinese medicine (TCM) [1]. Furthermore, safflower is also an important oil crop grown in multiple countries [2–5]. The safflower seeds have a high content of polyunsaturated fatty acids (PUFAs) with a high proportion of linoleic acid (LA). Linoleic acid is protective against various conditions such as cardiovascular and autoimmune diseases. It is also used as a precursor in the biosynthesis of arachidonic acid, prostaglandins, leukotrienes and thromboxane, which have extensive medicinal and nutritional values [6, 7].

Polyunsaturated fatty acids are important nutrients and cannot be synthesized in human body [8]. At present, improving the nutritional quality of safflower seeds is a vital objective for oil-utilized safflower breeding. The increased efficiency and decreased cost of RNA sequencing (RNA-seq) makes thorough investigation of the metabolite mechanisms possible. In flaxseed [9], peanut [10], olive [11], oil palm [12] and soybean [13], the profiling of differentially expressed genes and the key genes involved in some metabolite variations have been identified using de novo transcriptome sequencing. For safflower, to date, complete genome information is not available, and most safflower researchers have concentrated on studies of the molecular mechanisms of the flowers [14] and different tissues [15], including the flowers, leaves and roots involved in flavonoids compounds biosynthesis. Although a large number of target genes information exists in the abovementioned datasets that can be utilized, a systematic transcriptome analysis of the dynamic developmental progress of safflower seeds is still lacking. Thus, it is essential to explore the expression of RNA transcripts at the whole gene expression level in safflower seeds to further the study above the molecular mechanism of polyunsaturated fatty acids biosynthesis.

In this study, the fatty acid compositions in the developing safflower seeds and several key stages concerning the accumulation of fatty acids were selected and used for the transcriptome analysis by Illumina sequencing technology. In total, 29,699 (length > 500 bp) and 15,817 (length > 1000 bp) unigenes were obtained from twelve transcriptomes of safflower seeds within four development stages. The assembly and annotation of the transcriptome data and gene expression profiles will lay a vital foundation for investigating the PUFA and TAG biosynthesis pathways. It also offers a new understanding of using genetic engineering technology to increase the LA content in crops.

## Results

### Lipid accumulation at different stages of seed development

**Fig. 1:**
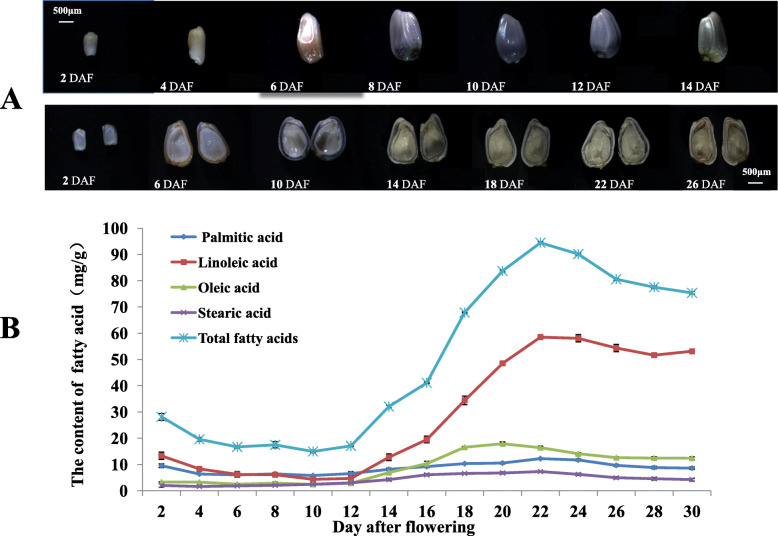
Observation and measurement of fatty acids across the developmental period of the safflower seeds. Note: **a** The developmental process of the safflower seeds (the up panel and the down panel respectively refer to the external and longitudinal section characteristics of the seeds). Ovaries were colected at 2 DAF (immature stage), and then every 2 days until 28 DAF (mature seeds). Because no obvious changes were observed for the external characteristics of the seeds after 14 DAF, 18, 22 and 26 DAF are not shown in the up panel. The scale of all figures is the same. **b** The four leading fatty acids at fifteen time points during safflower seed development (mean ± SD, *n* = 3)

Safflower seeds from different developmental stages were quickly frozen at − 80 °C. Fatty acid composition and content were analysed by gas chromatography-mass spectrometry (GC-MS) at fifteen different developmental stages, referring to the methodology of Li et al. and Guan et al [16, 17] As revealed in Fig. 1, there were four leading components, particularly, linoleic acid (LA: C18:2^Δ9c, 12c^), oleic acid (OA: C18:1^Δ9c^), palmitic acid (PA: C16:0) and stearic acid (SA: C18:0). The linolenic acids (ALA: C18:3^Δ9c, 12c, 15c^) were only detected during 2 ~ 10 DAF during the seed developmental progress, and their content was extremely low and was therefore ignored. The accumulation of the major fatty acids was detected during seed development, and the results showed that fatty acids content continuously changed until the seeds matured.

The seed development progress could be compartmentalized into three periods. In the initial period (2 ~ 10 DAF), the seeds gradually expanded, and their fatty acids content was low; after 10 DAF, however, a period of rapid fatty acid accumulation (10 ~ 22 DAF) appeared, along with dehydration phenomenon. Then, the fatty acid content subsequently declined as the seeds approaching full maturation (22 ~ 30 DAF). The total fatty acids in the safflower seeds gradually increased after 10 DAF and reached a maximum of 94.474 mg/g at 22 DAF. Then, it had a slight decrease after 22 DAF until maturity. The unsaturated fatty acids (LA and OA) were mainly increased at 10–22 DAF (Fig. 1b). In addition, an extraordinary high LA level was observed as the seeds matured, as shown in Fig. 1b. The LA content increased from 4.68 to 58.52 mg/g. However, the other major fatty acids were maintained at relatively low levels.

### Illumina sequencing and de novo assembly

To investigate the molecular mechanism of the seeds development and lipids accumulation in safflower, twelve cDNA libraries including three biological repeats were constructed from four time points during the seed development depending on the fatty acid content (i.e., the initial point 10 DAF, two total fatty acid accumulation points 14 and 18 DAF, and the highest content point 22 DAF). The cDNA libraries were processed using the high-throughput sequencing platform. The data were analysed at the four time points and the correlation analysis was detected with the Pearson correlation coefficient. The correlation coefficient, ranging from 0.928 to 0.997, indicated high reproducibility between duplicate samples. As Table 1 shows, the raw and clean reads of each sample were obtained, and the clean reads were selected and submitted to the National Centre for Biotechnology Information (NCBI) Short Read Archive (accession number: SRP186527). The percentage content of libraries were 47.27, 47.31, 47.23% (10 DAF), 47.26, 48.81, 48.12% (14 DAF), 50.44, 49.90, 50.76% (18 DAF), 50.50, 49.60, and 50.67% (22 DAF), respectively (Table 1).

**Table 1:** Summary of safflower seed transcriptome data sequenced by Illumina platform

	10d-1	10d-2	10d-3	14d-1	14d-2	14d-3	18d-1	18d-2	18d-3	22d-1	22d-2	22d-3
Raw reads	48,966,050	49,258,088	49,423,638	48,859,224	48,689,620	49,464,698	49,244,546	49,660,394	49,848,840	49,010,116	49,436,058	49,462,172
Clean reads	47,233,772	47,454,588	47,702,950	46,961,678	47,095,500	47,747,890	47,608,154	47,761,832	48,160,186	47,029,020	47,463,002	47,624,314
GC percentage	47.27%	47.31%	47.23%	47.26%	48.81%	48.12%	50.44%	49.90%	50.76%	50.50%	49.60%	50.67%

**Fig. 2:**
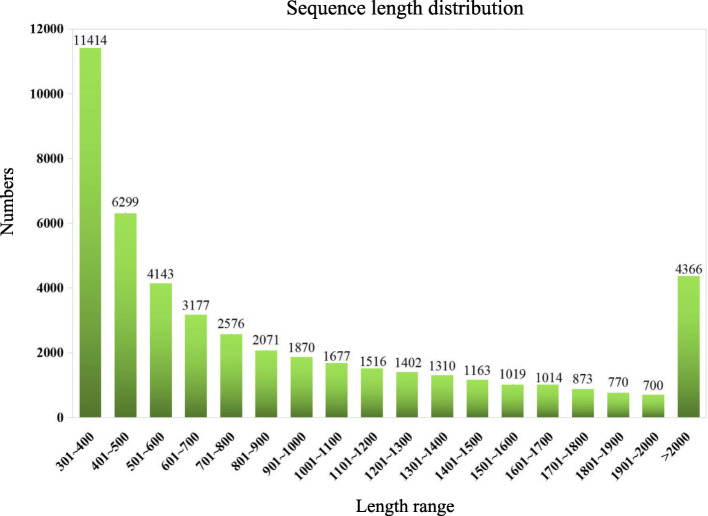
Size distribution of safflower Illumina reads. Note: The abscissa represents sequence length range, the unit is “bp”, and ordinate represents the number of sequences located in the corresponding length range

Trinity was used to assemble these transcriptome data without a reference genome sequence [18, 19]. Only the E-value of reads lower than 1e-10 with coverage higher than 80% were selected and used for subsequent analysis. In total, 47,360 non-redundant unigenes with a mean length of 948 bp ranging from 301 to 11,431 bp were obtained, and the size distribution of the unigenes revealed that 15,817 unigenes (33.40%) were longer than 1000 bp (Fig. 2).

### Gene functional annotations

For the 47,360 non-redundant unigenes, possible coding sequences (CDS) and their derived amino acid sequences were annotated. BLAST searching was performed (E-value ≤10^− 5^) against several public databases such as the NCBI non-redundant protein sequences (NR), Kyoto Encyclopedia of Genes and Genomes (KEGG) and Gene Ontology (GO) databases [20]. We found that 33,751 (70.00%), 25,464 (53.77%), 12,735 (26.89%), 19,246 (40.64%), 30,634 (64.68%), 22,689 (47.91%), and 99 (0.21%) of the safflower unigenes were consistent with sequences in the NR, Swiss-Prot, KOG, KEGG, GO and Pfam databases, respectively. Likewise, the species with the optimal match for each gene were 68.55% with *Cynara cardunculus* var. *scolymu*s, 3.37% with *Homo sapiens*, 2.1% with *Daucus carota subsp. sativus*, and 2.08% with *Vitis vinifera* L. (Additional file 1: Fig. S1), and these results were aligned with recent reports on the flower transcriptome analysis of safflower [14, 15, 21].

**Fig. 3:**
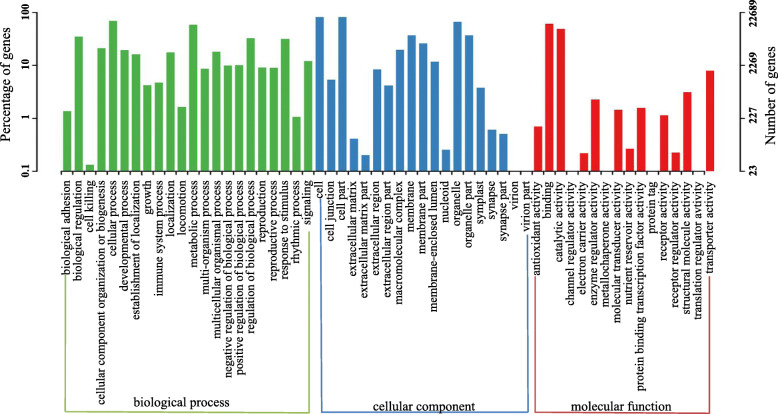
Functional classification of GO annotation of safflower unigenes. Note: Unigenes were assigned to three categories: biological processes, cellular components, or molecular functions

Gene ontology (GO) assignments were used to sort the unigenes functions. The cellular component category included the greatest number of unigenes (20,038), followed by molecular function (19,321) and then biological process (19,081). Within the cellular component category, “cell” “cell part” and “organelle” were the most enriched, and protein involved in “cellular process, metabolic process” and “biological regulation” was enriched in the biological process category. For molecular function, “binding” and “catalytic activity” accounted for most of the unigenes (Fig. 3).

To further evaluate the effectiveness of the functional annotation, KOG classifications were used to select the previously annotated unigenes. In total, 19,246 of the 47,360 sequences had a KOG classification (Additional file 1: Fig. S2), and they were divided into 25 KOG categories. Cluster “general function prediction only” showed the largest group (4218; 21.92%) followed by “signal transduction mechanisms” (2149; 11.17%) and “posttranslational modification, protein turnover and chaperones” (2093; 10.87%) while the categories of “cell motility” (17; 0.08%) represented the smallest group, which verified that the safflower seed development was associated with complex molecule regulatory processes.

Kyoto Encyclopedia of Genes and Genomes is believed to provide a platform for thorough analyses of gene function about the networks of gene expression products [22]. Among the 47,360 annotated unigenes, 11,632 were assigned into 24 KEGG pathways to identify the functioned biological pathways. Five main categories were initiated in the 24 pathways, and the three typical pathways were “signal transduction” with 1524 members, “Translation” with 1093 members, and “Carbohydrate metabolism” with 979 members. Furthermore, 15,929 unigenes with annotation could be classified into 215 KEGG pathways. Genes about “Ribosome” were the most abundant (403), followed by “Carbon metabolism” (367) and “Protein processing in endoplasmic reticulum” (356) (Additional file 1: Table S1).

It should be specially mentioned that 563 unigenes belonged to the lipid metabolism category and might play critical roles in fatty acid biosynthesis and metabolism. Among the 563 unigenes, 51 and 55 unigenes were classified into the “FAs biosynthesis” and “biosynthesis of USFAs” sub-pathways, respectively, which were more likely to participate in lipid biosynthesis for safflower seeds. In addition, LA metabolism (29 unigenes), glycerolipid metabolism (92 unigenes), fatty acid elongation (52 unigenes) and glycerophospholipid (145 unigenes) were also found (Additional file 1: Table S1). The above mentioned unigenes for lipids metabolism were important for the characterization of key enzyme genes associated with USFA and TAG biosynthesis in safflower seeds.

Researchers found that switching carbon involved in starch and oil biosynthesis by controlling genes involved in oil and starch biosynthesis in transgenic *Arabidopsis thaliana* can raise the energy density of the vegetative tissues [23]. Similarly, over 1500 unigenes participating in lipid metabolism were addressed to FAs synthesis and TAG assembly during *Arachis hypogaea* seed development [1, 10]. For the two edible oil crops, a difference clearly exists between safflower and *Arachis hypogaea* in their oil content (30% versus 50%, approximately, dry seed) and predicted gene number involved in lipid biosynthesis (563 versus 1500, respectively). This difference revealed that different oil biosynthesis mechanisms exist in the two oil crops. In addition, 293 and 205 unigenes, among the 979 carbohydrate metabolism unigenes, were classified into the “starch and sucrose metabolism” and “glycolysis/gluconeogenesis” sub-pathways, respectively.

### Analysis of differentially expressed genes during safflower seed development

Differentially expressed genes (DEGs) offer traces related to the molecular events of seed development process. The data were collected at different time points, and time-series differential expression analyses were performed to explore the global temporal patterns of transcriptomic changes, paying attention to the dynamic changes of FA biosynthesis and degradation. The DEGs across a time series were defined as genes that are differentially expressed between any two time points. Seeds at 10 DAF were set as the control, and 9780, 16,731 and 18,165 DEGs (*p*-value< 0.05 and fold change > 2) were identified at 14, 18 and 22 DAF, respectively. Seeds at 14 DAF were set as the control; a total of 10,894 and 13,629 DEG were identified at 18 and 22 DAF, respectively. Seeds at 18 DAF were set as the control; a total of 4227 DEGs were identified at 22 DAF (Additional file 1: Table S2). During the development of the safflower seed, there were 503, 587 and 1685 up-regulated unigenes at 10 DAF vs. 14 DAF, 14 DAF vs. 18 DAF and 18 DAF vs. 22 DAF, respectively, while 604, 574 and 634 unigenes were down-regulated (*p* value< 0.01).

Further analysis of the DEGs may identify the ones related to the biosynthesis and accumulation of lipids during the development of safflower seeds. The DEGs with a GO annotation were further classified into several subsets to analyse the potential functions of the genes with significant expression level changes at the transcriptional level among the three contrasting groups. According to the categories, the three compared groups showed similar patterns (Additional file 1: Fig. S3).

The KEGG functional enrichment analysis was then performed to reveal their biological functions. Within contrast group 10 DAF vs. 14 DAF, the different unigenes were mainly enriched in ribosome (137 unigenes), plant hormone signal transduction (106), starch and sucrose metabolism (95), carbon metabolism (79), and biosynthesis of amino acids (74). In the contrast group 14 DAF vs. 18 DAF, the most represented KEGG pathways were ribosome (202), carbon metabolism (105), biosynthesis of amino acids (96), plant hormone signal transduction (94), and starch and sucrose metabolism (85). Within contrast group 18 DAF vs. 22 DAF, the most represented KEGG pathways were pI3K-Akt signalling pathway (39), phagosome (38), biosynthesis of amino acids (32), focal adhesion (31), and starch and sucrose metabolism (30) (Additional file 1: Table S3). The above analyses revealed that the ribosome, biosynthesis of amino acids, starch and sucrose metabolism and carbon metabolism activity underwent enhancement with safflower seed development from 10 DAF to 18 DAF. In the contrast group 18 DAF vs. 22 DAF, the number of different unigenes enriched in ribosome, biosynthesis of amino acids and carbon metabolism pathway were significantly lower than the other contrast groups.

These results indicated that safflower seeds have vigorous life activities between 10 DAF to 18 DAF, and then gradually enter a dormancy period after 18 DAF. In addition, it is necessary to mention the unigenes in the pathways responsible for seed oil biosynthesis. Fatty acid metabolism activity was mainly enriched from 10 DAF to 18 DAF, and the number of different unigenes decreased in the contrast group 18 DAF vs. 22 DAF. For LA metabolism, the largest number of different unigenes emerged in the contrast group 14 DAF vs. 18 DAF (Additional file 1: Table S4). These findings were consistent with the results that the contents of total fatty acids and LA sharply increased from 14 DAF to 18 DAF in safflower seed. These analyses may lay an important foundation for the identification of candidate genes associated with LA biosynthesis.

### Analysis of DEGs involved in lipid metabolism

**Fig. 4:**
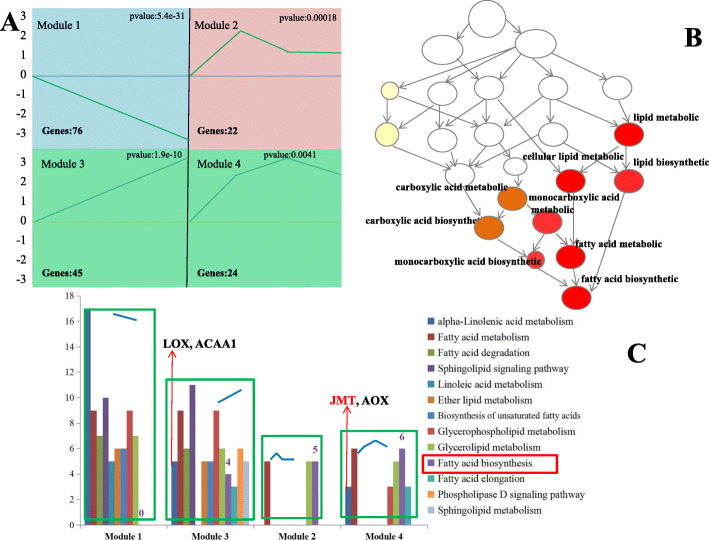
The analysis of differentially expressed genes related to lipid metabolism. Note: (A) The normalized average expression level of DEGs in each module at 10, 14, 18, and 22 DAF. (B) The directed acyclic graph of GO analysis in module 4. The circles filling with different colours represent the difference of gene enrichment (The darker the colour, the higher degree of enrichment) (C) The KEGG pathway analysis in four modules. Different colours represent the different enrichment pathways. The Y-ais represents the number of enriched genes. LOX: lipoxygenase, ACAA1: acetyl-CoA acyltransferase 1, JMT: jasmonate O-methyl-transferase, AOX: allene oxide synthase

All DEGs related to lipid metabolism were selected and analysed using GCBI (https://www.gcbi.com.cn) online software. Four significant modules were found and named as module 1 (down-down-down), module 2 (up-up-up), module 3 (up-down-no change) and module 4 (up-up-down) (Fig. 4a), respectively. Gene ontology enrichment analyses revealed that the largest number of significant enrichments related to fatty acid/lipid metabolism existed in module 4 (Fig. 4b). Figure 4c shows the results of the KEGG pathway analyses; module 3 contained the largest number of pathways enrichments (12/13), while the least number of pathways enrichments (3/13) existed in module 2. The fatty acid biosynthesis pathway existed in all modules except for module 1, suggesting that most genes related to the FAs biosynthesis process were up-regulated from 10 DAF to 14 DAF.

The ALA content was extremely low throughout the entire safflower seed developmental progress, and disappeared after 10 DAF. We observed that 17 genes related to α-linolenic acid metabolism were present in module 1, while only 5 and 4 genes existed in modules 3 and 4, respectively. The expression patterns were similar in module 3 and module 4, and lipoxygenase (LOX), acetyl-CoA acyltransferase 1 (ACAA1), jasmonate O-methyl-transferase (JMT), and allene oxide synthase (AOX) were upregulated from 10 DAF to 18 DAF. These results suggesting the low amount of α-linolenic acid synthesized before 10 DAF might be mainly used as a precursor substance for the biosynthesis of methyl jasmonate (MeJA) rather than being used for other metabolic processes, and the α-linolenic acid content might have been provided by the maternal tissues before the embryo began to form at 10 DAF. In total, our detailed results provide helpful insights for the identification of key genes related to lipid metabolism.

### Identification of genes related to FAs and TAGs biosynthesis in safflower seeds

**Fig. 5:**
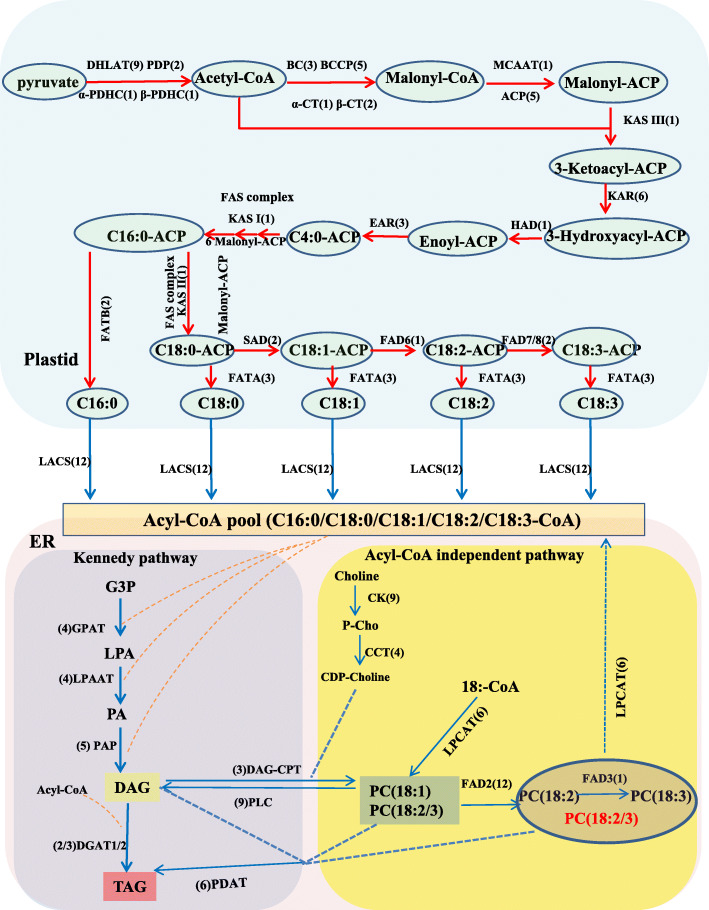
The de novo fatty acid and triacylglycerols biosynthetic pathways in safflower seeds. Note: The red and blue arrows represent the biosynthesis pathway in the plastid and ER, respectively. Lipid substrates are abbreviated: C16:0, palmitic acid; C18:0, stearic acid; C18:1, oleic acid; C18:2, linoleic acid; C18:3, linolenic acid. Enzyme/protein abbreviations are: DHLAT, dihydrolipoamide acetyltransferase; α-PDHC, pyruvate dehydrogenase alpha subunit; β-PDHC, pyruvate dehydrogenase beta subunit; PDP, pyruvate dehydrogenase phosphatase; α-CT, carboxyl transferase alpha subunit; β-CT, carboxyl transferase beta subunit; BC, biotin carboxylase; BCCP, biotin carboxyl carrier protein; MCAAT, malonyl-CoA ACP transacylase; ACP, acyl carrier protein; KAS I, II, III, ketoacyl-ACP synthase I, II, III; KAR, ketoacyl-ACP reductase; HAD, hydroxyacyl-ACP dehydrase; EAR, enoyl-ACP reductase; SAD, stearoyl-ACP desaturase; FAD6, oleate desaturase (chloroplast); FAD7/8, linoleate desaturase (chloroplast); FAD2, oleate desaturase; FAD3, linoleate desaturase; FATA/B, acyl-ACP thioesterase A/B; LACS, Long-Chain Acyl-CoA Synthetase; GPAT, glycerol-3-phosphate acyltransferase; LPAAT, 1-acylglycerol-3-phosphate acyltransferase; PAP, phosphatidic acid phosphatase; DGAT1/2, acyl-CoA: diacylglycerolacyltransferase 1/2; PLC, phospholipase C; CK, choline kinase; CCT, choline-phosphate cytidylyltransferase; LPCAT, lysophosphatidylcholine acyltransferase/lyso-PAF acetyltransferase; PDAT, phospholipid:diacylglycerolacyltransferase; DAG-CPT, diacylglycerol cholinephosphotransferase

Based on the previous research involving the FAs and TAGs biosynthetic pathways, most of the enzymes participating in lipid biosynthesis have been identified [8, 10]. Two hundred fifty five unigenes associated with FAs and TAGs biosynthesis were found (Additional file 2: Table S5). The biological functions of the genes were clarified by pathway-based analyses. We found 80 genes participating in the initiation and acyl chain elongation steps for de novo FA biosynthesis (Additional file 2: Table S5). These genes had one or more isoform, and their expression levels were higher at 10 DAF or 14 DAF, and lower at 18 DAF or 22 DAF, suggesting that they were mainly responsible for the beginning of FA biosynthesis during the early safflower seed developmental stages (Additional file 1: Fig. S4). The first reaction is catalysed by acetyl-CoA carboxylase (ACCase), and it generates malonyl-CoA. ACCase is a rate-limiting enzyme which controls the rate of fatty acid synthesis; subsequently, repeated condensations of malonyl-ACP with the growing acyl-ACP chain are derived by FAs synthase, continuously adding two carbon units until forming 16:0-ACP. The newly synthesized FAs are mainly exported to the ER as acyl-CoA and join the glycerolipid synthesis pathway in oilseeds [24] (Fig. 5).

**Fig. 6:**
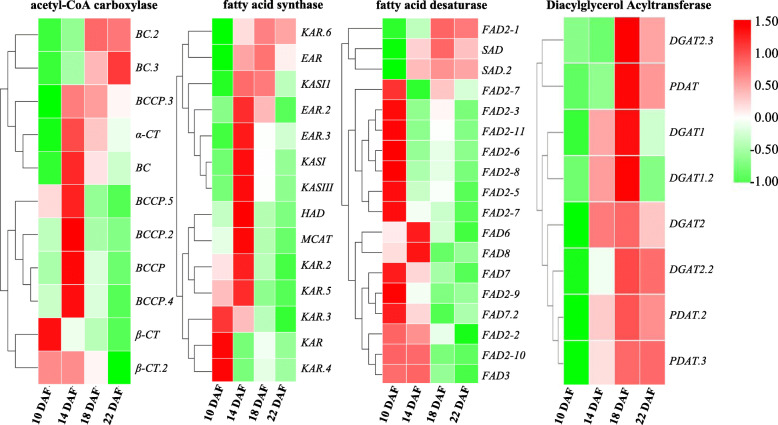
The heat map analysis of key enzyme genes related to FAs biosynthesis among the different safflower seed developmental stages. Note: Abbreviated lipid substrates are the same as in Fig. 5. Different colours represent different normalized FPKM values

Genes encoding ACCase and FAS had the highest expression level at 14 DAF (Fig. 6), suggesting that de novo synthesis of FAs in plastids was active during 10 to 14 DAF, and many acetyl-CoA products (16-C or 18-C) were exported to the endoplasmic reticulum (ER) for TAG assembly. In addition, 12 unigenes encoding long-chain acyl-CoA synthetases (LACS) were identified, and 7 unigenes had high expression levels at 10 DAF and then decreased, while 5 unigenes were maximally expressed at 14 DAF. The different expression patterns of *LACS* unigenes might suggest that different substrates are catalysed by the *LACS* gene family and determine the composition of the ER acyl-CoA pool (Additional file 2: Table S5).

Triacylglycerols are the major components of storage lipids in plants and can be synthesized through the Kennedy pathway [25]. We identified 57 unigenes for the Kennedy pathway and acyl editing/alternative TAGs synthesis process in safflower seeds (Additional file 2: Table S5). Triacylglycerols can also be synthesized in plants mainly via two different acyl-CoA independent pathways, both of which exist in safflower seeds, catalysed by diacylglyceroltransacylase (DGAT) and phospholipid: diacylglycerolacyltransferase (PDAT) during the last steps of the acyl-CoA independent pathways. Several DGAT and PDAT isoforms were expressed highly at 18 DAF, revealing that TAGs were efficiently synthesized in this period and that the partial *PDAT* genes have a higher expression level, suggesting that this pathway might also be important in the safflower seed developmental progress (Figs. 5 and 6). In addition, 42 unigenes encoding phospholipases were also identified (Additional file 2: Table S5). Some reports revealed that lipases might participate in the remodelling of TAGs after synthesis in castor bean [26, 27]. In total, 4 unigenes encoding triacylglycerol lipase were found, and the function of these genes in safflower seed developmental should be further studied. These identified genes would supply vital clues to clarify further the molecular mechanisms of safflower seeds oil accumulation.

Researchers previously revealed that most of the FAs formed in plastid were available for TAGs biosynthesis after desaturation [8]. For the process of fatty acids desaturation, 18 unigenes for fatty acid desaturase (FAD) were identified in safflower seeds. Among these unigenes, 2 stearoyl-ACP desaturase (SAD) and 13 oleate desaturase genes (11 *FAD2* and one *FAD6*) were found. Different from other plants that have a high ALA content in oilseed, only 4 unigenes encoding omega-3 fatty acid desaturase (one for *FAD3*, two for *FAD7* and one for *FAD8*) were identified (Additional file 2: Table S5). It is worth mentioning that the ω-6 (*FAD6*) and ω-3 (*FAD7*) fatty acid dehydrogenase genes localized in plastids had extremely low expression levels throughout the safflower seed developmental progress. The reason for these low levels might be that the lower contents of 18:2-CoA and 18:3-CoA were exported to the acetyl-CoA pool at 10 DAF.

The *FAD2* genes (located in the ER, except *FAD2–1*) were expressed mainly at 10 DAF, and decreased sharply after 10 DAF (Fig. 6). Although the *FAD2* gene family is enormous in safflower, some copies were identified as having no functions or a lower activity of oleic acid desaturase [28]. Only one *FAD3* gene (located in the ER) was identified as LA desaturase, and its expression level was extremely low throughout the seed development process, which might cause the low content of LA and the extremely low content of ALA detected at 10 ~ 12 DAF.

However, the *FAD2–1* gene and the SAD genes had their highest expression levels at 18 DAF, especially for the *FAD2–1* gene (Additional file 2: Table S5 and Fig. 6), suggesting during 14 ~ 18 DAF, most 18:0-ACP was transformed to 18:1-ACP by *SAD* genes in the plastid, then 18:1-CoA was transformed to 18:2-CoA by the *FAD2–1* gene in the ER, and because of the extremely low transcript level of *FAD3*, 18:2-CoA could not be further transformed to 18:3-CoA, which might result in an abundant accumulation of LA in TAGs. In previous research by our group, the *FAD2–1* gene was thought to be the key gene that controlled the LA content in mature safflower seeds [29, 30], and the transcriptome library of safflower developing seeds was coincident with our previous research. Although the function of the *FAD2–1* gene has been studied, the regulatory mechanism of how *FAD2–1* is highly expressed at 18 DAF has not been explored. The research into the molecular regulative mechanism of the abundant accumulation of LA is important for purposefully altering the proportions of OA and LA in safflower oilseed using gene editing technology.

### Identification of the possible transcription factors involved in regulating FAs and TAGs biosynthesis and embryonic morphogenesis

**Fig. 7:**
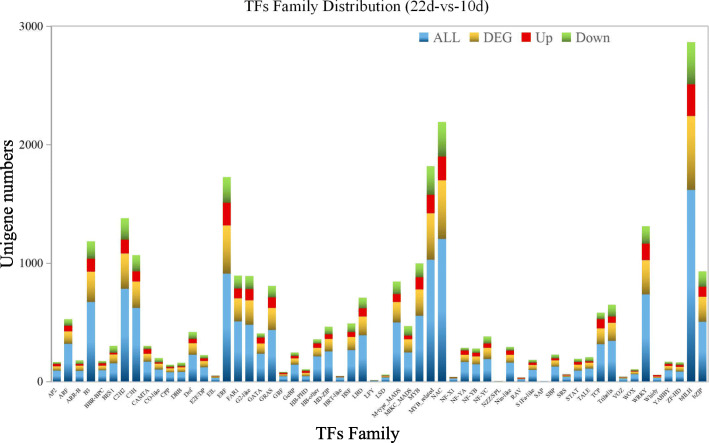
The different transcription factor family distributions between 10 to 22 DAF. Note: “Up” represents the upregulated genes, “Down” represents the downregulated genes, “DEG” represents the differentially expressed genes, and “All” represents the total genes

Among the seed developmental processes, 58 TFs families were found, and the top 5 different TFs families were the *bHLH*, *NAC*, *MYB*-related, *ERF* and *C2H2*-domain containing families. The results revealed above mentioned the TFs might be important for the seed developmental progress. In addition, the major upregulated differentially expressed transcription factors (log2 Fold Change> 3) were identified between the 10 to 22 DAF seeds (Fig. 7). The results of KEGG enrichment analyses of the DEGs, combined with the de novo fatty acid and TAGs biosynthetic pathways in safflower developing seeds and the expression pattern analyses of key enzymes related to FAs biosynthesis during the safflower seed developmental process (Figs. 5 and 6) revealed that fatty acid biosynthesis and glycolysis mainly occurred at 10–14 DAF, the biosynthesis of unsaturated fatty acid occurred in 14–18 DAF, and during 18–22 DAF, seed dormancy was the dominant cellular process.

**Fig. 8:**
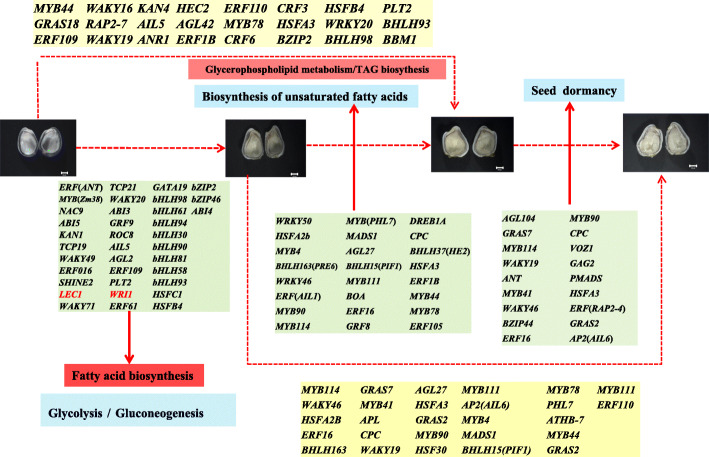
The transcription factors (TFs) involved in regulating FAs, TAGs biosynthesis and embryonic morphogenesis. Note: The arrows represent those candidate TFs that regulate the corresponding biological process, respectively. The TFs (log2FoldChange > 3, *p* < 0.01) were chosen for candidate TFs between the contrast groups

As shown in Figs. 8, 36 TFs that might be associated with fatty acid biosynthesis and glycolysis were selected, including 8 basic helix-loop-helix (*bHLH*) domain-containing proteins, 8 *AP2/ERF* domain-containing proteins, 2 HSF-type proteins, 3 *WRKY* transcription factors, 3 ABA response genes (*ABI3*, *ABI4* and *ABI5*), and others. These revealed that *bHLH* and *AP2/ERF* domain-containing proteins were important for seed development. We identified 24 TFs that might be involved in USFAs and TAGs biosynthesis, including 6 *MYB*, 2 *bHLH*, 2 *MADS-box*, 2 *HSF-type* transcription factors and 3 ethylene-responsive transcription factors, among others. *MYB* transcription factors and ethylene-responsive transcription factors could take part in the response to drought and dehydration, and this participation was observed when the safflower seeds underwent severe dehydration during 10–18 DAF. In addition, 18 TFs that might be involved in seed dormancy were identified. The TFs (*LEC1* and *WRI1*) marked by red in Fig. 8 can regulate fatty acid biosynthesis as reported in previous research [31–33]. In addition, the *bZIP* transcription factor (*Gmbzip123*) can promote the expression of genes involved in sucrose transporter and cell-wall invertase by binding to their promoters and enhancing the lipid content [34]. *Atbzip67* can regulate the gene expression level by binding to the promoters of the *FAD3* gene and enhance omega-3 fatty acids content [35]. The transcription factor *AGL1* (MADS-box family) can promote the development of somatic embryos and change the content of the oils [36]. The identifications of TFs involved in FAs and USFAs biosynthesis has provided additional important clues about fatty acids biosynthesis.

### Identification of the TFs related to the regulation of *FAD2–1* gene expression

**Fig. 9:**
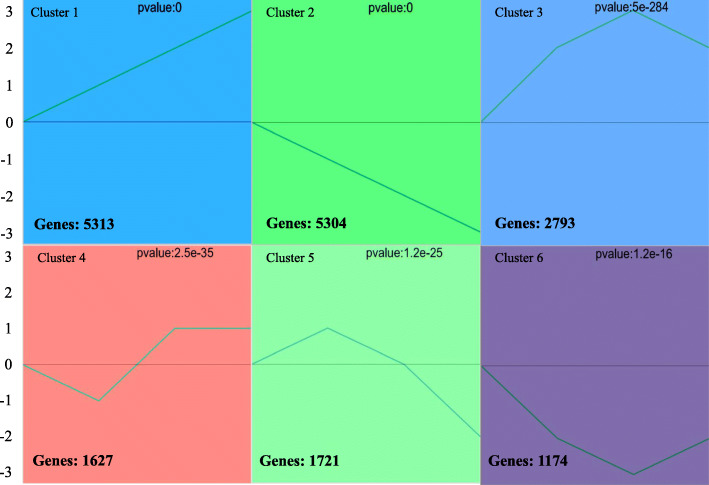
Differentially expressed genes (DEGs) in transcriptomic analysis. Note: The normalized average expression levels of DEGs in each cluster at 10, 14, 18, and 22 DAF

**Fig. 10:**
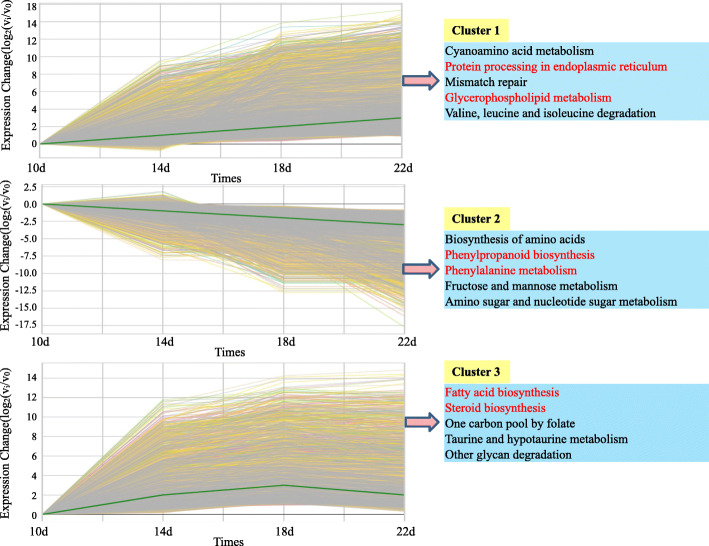
The KEGG enrichment analysis (top 5) among four seed developmental time points in three typical clusters

To identify the TFs involved in regulating the expression of the *FAD2–1* gene, the series test of cluster (STC) analysis was executed among all unigenes obtained from the transcriptomic data. The STC analysis was executed by GCBI (https://www.gcbi.com.cn) online software and 27 clusters including 6 significant clusters were found (*P* < 0.05) (Fig. 9). Six clusters were found to better reflect the expression patterns of DEGs. All of these clusters and the corresponding gene members are shown in Fig. 9. The genes have similar temporal expression patterns in each cluster and may be involved in the same biological process. The time interval was divided into three stages: 10–14, 14–18, and 18–22 DAF. Six broad classes become apparent across these stages: “up-up-up” (cluster 1), “down-down-down” (cluster 2), “up-up-down” (cluster 3), “down-up-no change” (cluster 4), “up-down-down” (cluster 5), and “down-down-up” (cluster 6) (Fig. 9). GO enrichment analysis revealed that only cluster 1 was significantly enriched in “seed development (27 unigenes)” and “seed oil body biogenesis (4 unigenes)”. Further KEGG enrichment analysis indicated cluster 1 was significantly enriched in “glycerophospholipid metabolism (18 unigenes)” (Fig. 10).

In total, among the 6 significant clusters, 3 representative key clusters were identified. In cluster 1 (up-up-up), 5313 unigenes were contained and the top 5 clusters with higher enrichment factors and smaller *p*-values by KEGG enrichment analysis were identified, including “protein process in ER”, “glycerophospholipid metabolism” and others. In contrast, in cluster 2 (down-down-down), 5304 unigenes were contained and the top 5 clusters with higher enrichment factors and smaller p-values by KEGG enrichment analysis were identified, including protein process in “phenylpropanoid biosynthesis”, “fructose and mannose metabolism” and others, revealing the conversion from sugar metabolism to lipid metabolism during the seed developmental progress. Because of the similar pattern to cluster 1, cluster 3 (up-up-down) was also selected for further analysis and 2793 unigenes were contained. The “fatty acid biosynthesis (8 unigenes)”, “fatty acid metabolism (9 unigenes)” and “steroid biosynthesis (6 unigenes)” pathways were significantly enriched in this cluster (Fig. 10).

**Fig. 11:**
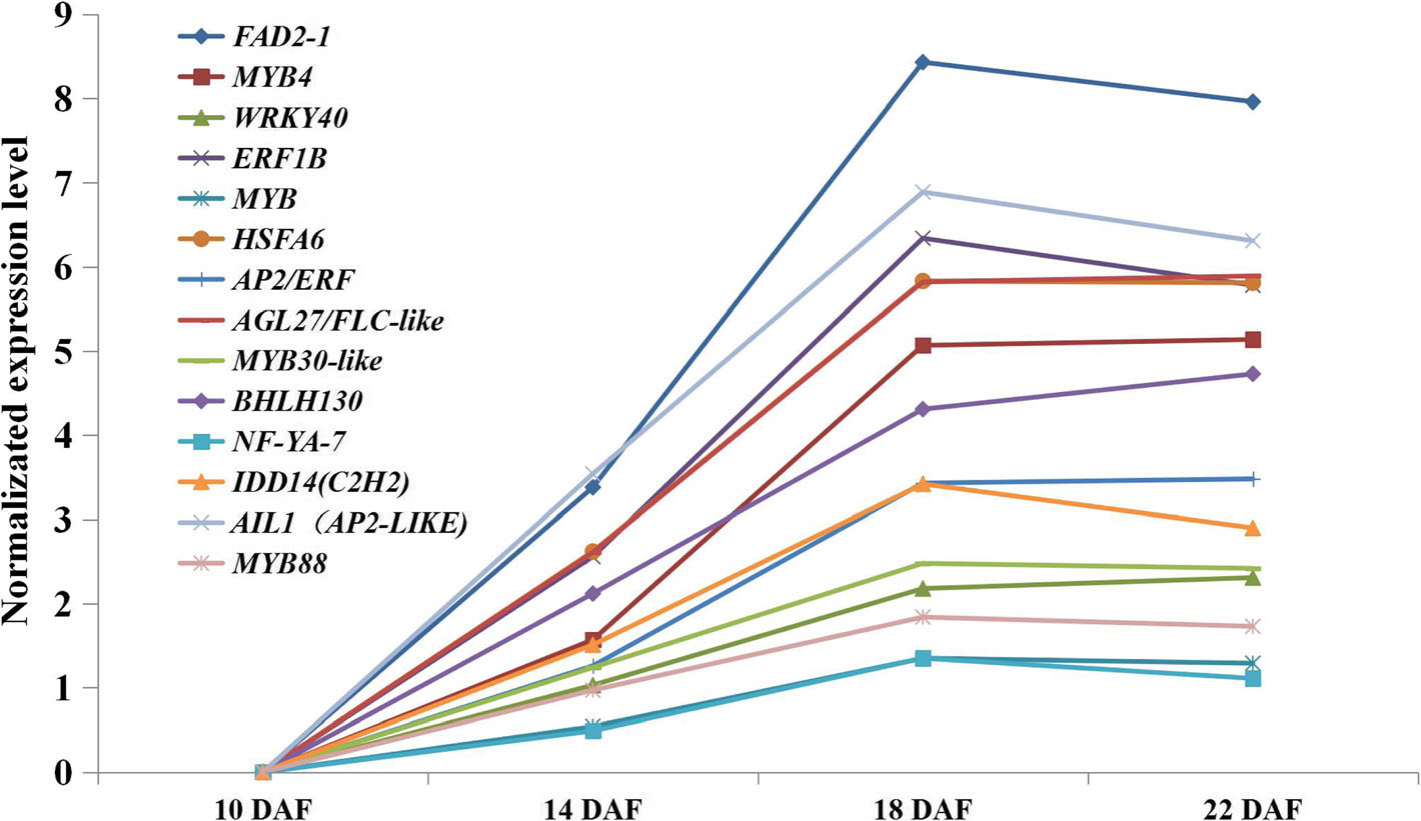
The normalized gene expression of the *FAD2–1* genes and 13 TFs that might be involved in regulating *FAD2–1* gene expression

The key enzyme gene *FAD2–1* (TRINITY_DN23030_c1_g7_i2_3) for LA synthesis was identified by BLAST analysis and the gene was found in cluster 1. Because of the similar expression patterns between cluster 1 and cluster 3, the 8106 genes in cluster 1 and cluster 3 were identified, and 201 TFs were identified for subsequent analysis. Pearson correlation analysis was performed using SPSS statistical analysis software, and 13 TFs were identified that had significant correlations (p-value< 0.01) with the *FAD2–1* gene (Fig. 11), and these TFs might participate in the regulation of the expression level of the *FAD2–1* gene. In our previous research (data unpublished), the 1500 bp sequence of the *FAD2–1* gene promoter was cloned and the cis-elements were predicted by the PlantCARE online software (http://bioinformatics.psb.ugent.be/webtools/plantcare/html/). The results revealed heat-stress response (HSE), GCC-box, low-temperature response (LTR) and E-box elements were contained in the *FAD2–1* gene promoter (Additional file 1: Fig. S5, Fig. S6). In addition, the prediction analysis was executed again by JASPER online software (http://jaspar.binf.ku.dk/) and the results revealed C2H2 zinc finger factors (Dof-type family), MADS-box family and ABF2 (bzip family) proteins were possible binding to the *FAD2–1* gene promoter and regulating the *FAD2–1* gene expression level.

### Real-time PCR analysis

**Fig. 12:**
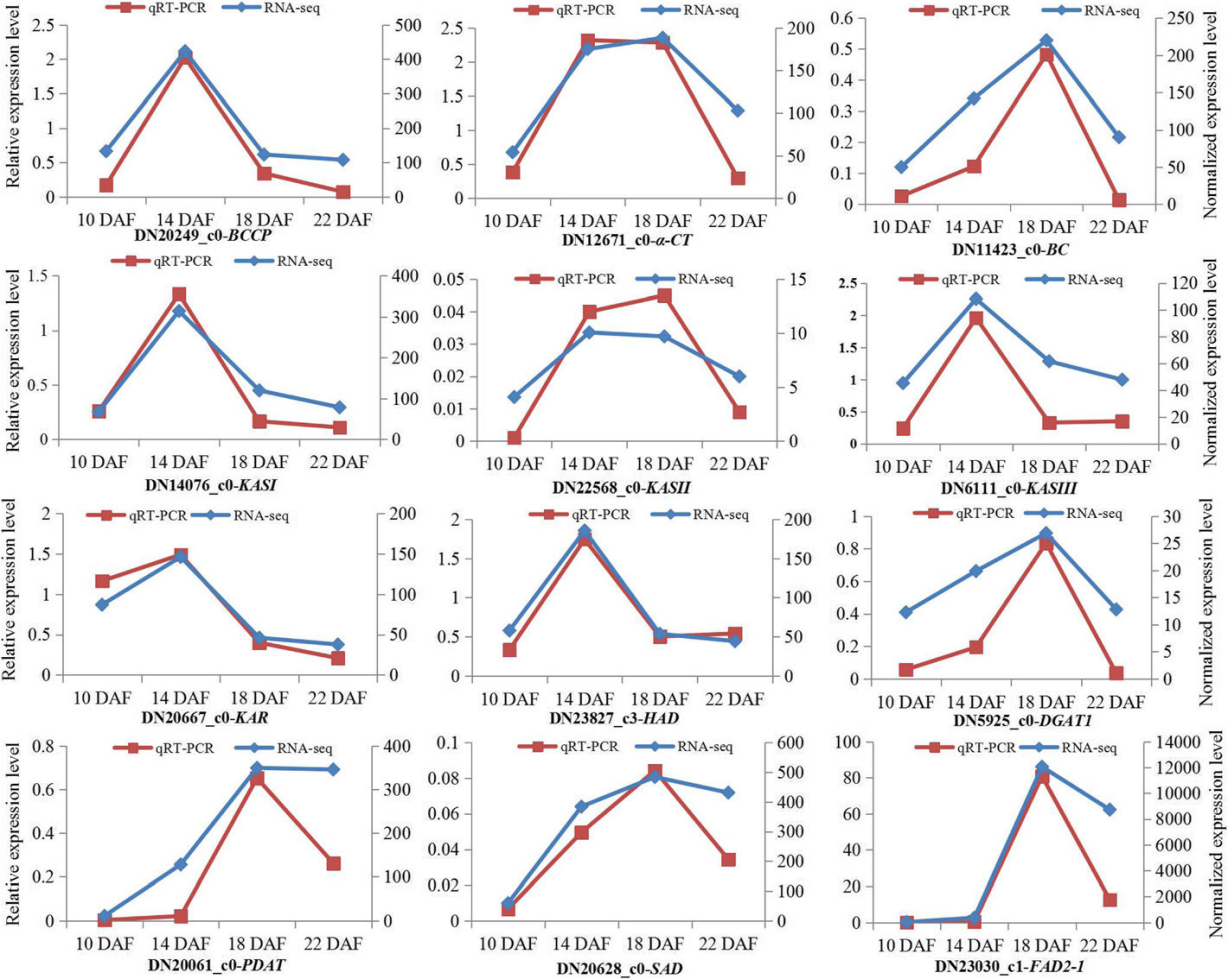
The expression levels of 12 genes at 10, 14, 18, and 22 DAF for qRT-PCR and the RNA-Seq experiment

To verify the temporal expression patterns of the DEGs obtained from the transcriptome data, 12 lipid-metabolism-related unigenes were selected for quantitative RT-PCR (qRT-PCR) analyses, which encoded three ACCases, 3-ketoacyl-ACP synthase I (KASI), 3-ketoacyl-ACP synthase II (KASII), 3-ketoacyl-ACP synthase III (KASIII), 3-ketoacyl-ACP reductase (KAR), 3-hydroxyacyl-[acyl-carrier-protein] dehydratase (HAD), diacylglycerol acyltransferase (DGAT1), phospholipid:diacylglycerol acyltransferase (PDAT), stearoyl-[acyl-carrier-protein] 9-desaturase (SAD), and microsomal omega-6 FA desaturase (FAD2–1). The same extracted RNA samples from the seeds at the four developmental phases were used as templates for RNA-Seq and qRT-PCR. The results between qRT-PCR and RNA-Seq for these 12 genes were basically analogous (Fig. 12). Five unigenes for *BCCP* (20249), *KASI* (14076), *KASIII* (6111), *KAR* (20667), and *HAD* (23827) showed an up-down-down expression pattern. The four unigenes for *DGAT* (5925), *PDAT* (20061), *SAD* (20628) and *FAD2–1* (23030) showed an up-up-down expression pattern. All of these 12 genes were observed to have a lower expression level in seeds at 22 DAF. Generally, the above results revealed that our transcriptome data were reliable for genes temporal expression analysis during the seed developmental processes in safflower.

## Discussion

Together with other PUFAs, LA plays a wide variety of roles in prophylactic effects and biological activities, and LA also has a vital role as a storage lipid and membrane lipid in plants [37, 38]. Because of the high value and importance of LA, its exploitation and utilization and investigations into the mechanisms of oil and PUFA biosynthesis have been extensively reported [10, 11, 39–42]. In recent years, safflower has been grown widely as a kind of traditional Chinese medicine (TCM) in China and as an ancient oilseed crop because of its high-quality esculent oil. Safflower oil is a critical nutritional supplement for specific populations, such as weanling infants and pregnant women.

The investigations of the molecular mechanisms involved in lipid metabolism are critical for genetic engineering of safflower to increase its oil accumulation level or to change the oil composition. In this research, transcriptome analysis was performed to reveal the temporal changes in FA biosynthesis during the seed developmental process. The temporal gene expression profiles were detected across three seed developmental stages, 10–14, 14–18, and 18–22 DAF. Our results showed that FA biosynthesis is a dominant cellular process during the first stage and the fatty acids desaturation mainly occurs at the first and second stages (oleate desaturation mainly in the second stage), and these results were in accordance with the changes of fatty acid contents across safflower seed development.

High levels of the transcripts of the ACCase gene and fatty acid synthase gene (FAS) at 10–14 DAF were detected. ACCase can catalyse the conversion of acetyl-CoA to malonyl-CoA, which is an irreversible but important reaction for the initiation of FA biosynthesis; ACCase is also thought to be a key enzyme that controls the rate of FA biosynthesis [43].

For the fatty acid desaturation, significant expression changes of two genes, stearoyl-[acyl-carrier-protein] 9-desaturase gene (*SAD*) from 10 to 14 DAF and oleate desaturase gene (*FAD2–1*) from 14 to 18 DAF, were observed by transcriptomic analyses, indicating that *SAD* and *FAD2–1* might be important for the synthesis of USFAs. Stearoyl-[acyl-carrier-protein] 9-desaturase is a key enzyme that can convert steroid-ACP to oleate-ACP, and oleate desaturase (FAD2–1) is also a key enzyme that can catalyse the conversion of monounsaturated fatty acids to diunsaturated fatty acids [28, 44]. In addition, only one isoform of the ER-located FAD3 enzyme was found, and its expression level was extremely low at 10–22 DAF, which was the primary reason for no detected ALA in mature safflower seeds. For TAG assembly, the *DGAT* genes were mainly expressed at 18 DAF, suggesting the progression of TAG assembly primarily occurred at the third stage (18–22 DAF). That result further revealed that in safflower, the FAs produced (the first stage) were not immediately available for TAG biosynthesis (the third stage) before desaturation (the second stage).

Many well-known genes involved in lipid metabolism were identified as *ACCase*, *FAS*, *DGAT*, *LACS* and others. Genes in a cluster with similar temporal expression patterns might have similar functions in the same cellular process, and thus except for the known genes, some of the remaining unknown genes in cluster 1 and 3 might also contribute to lipid biosynthesis or regulate the biosynthesis pathway. Our investigation provides vital clues for further exploring the potential roles of these genes in the fatty acid biosynthesis process. In addition, many potential novel candidate regulatory factors were also revealed, including some known TFs (*WRI1*, *LEC1*, and *ABI3*) that were all up-regulated in the first stage (10–14 DAF) [31–33]. Except for the known TFs, additional works are required to enlarge the pool of TFs for safflower in future researches. The above-mentioned candidate TFs might play important roles in lipid metabolism (FAs biosynthesis, FAs metabolism, FAs elongation and others).

## Conclusions

In this study, to provide comprehensive insights about the molecular basis of oil accumulation, the morphological characters, and the FA compositions and contents, we performed whole gene expression scanning in developing safflower seeds. An increased expression level of genes involved in most FAs biosynthesis showed a positive correlation with an early increase in seed oil content at the embryogenesis phase. During the early seed developmental process, the cells differentiated and expanded rapidly, and abundant membrane lipids were synthesized in large quantities. The genes involved in most FAs biosynthesis that were highly expressed during the early seed developmental progress were mainly responsible for the biosynthesis of membrane lipids in safflower seeds. Subsequently, for genes involved in FAs biosynthesis, a coordinated down-regulation appeared until the desiccation phase, while the genes involved in TAG biosynthesis were activated during the seed-filling phase until they achieved the maximum lipid storage in the seeds. Our research will provide a possible correlation between the transcriptional profile of some lipid-metabolism-related genes and the dynamic accumulation pattern of seed oil in safflower. Meanwhile, this study also contributes key clues for further illumination of the regulatory mechanism of oil accumulation.

## Methods

### Plant material

Seeds of the safflower cultivar (Chuan Hong No1) were collected in 2017 from the Wenjiang experimental field (black loam, sown in September and harvested in May, the permission to collect plant samples has been obtained), College of Agronomy, Sichuan Agriculture University, China, It had been grown under the same growing conditions for a continuous five years. The seed developmental process from flowering until maturation was observed and the seeds were collected at intervals of two days, including a range of 28 d, and the oil content was detected. Seeds from the four development stages (10, 14, 18 and 22 DAF) were collected used for the transcriptome, and three biological duplications were conducted for each development stage. The collected seeds were stored at − 80 °C.

### Fatty acid detection

Seeds at fourteen development stages (2, 4, 6, 8, 10, 12, 14, 16, 18, 20, 22, 24, 26, and 28 DAF) were transmethylated, extracted, and detected and analysed by GC-MS. [17]

### High throughput sequencing

Total RNA was extracted and purified from safflower seeds collected at 10, 14, 18 and 22 DAF, respectively, and RNA integrity, concentration and quality were detected using Huang et al.’s method [42]. The cDNA libraries were constructed and sequenced using Liu et al.’s method [45].

### De novo unigene assembly and annotation

Clean reads were assembled using Trinity software under Linux [46]. Unigenes were aligned using BLASTX (E-value<1e-5). Partial CDS sequences were predicted using ESTScan [47]. Functional annotations were performed according to Li et al.’s method [11].

### Differential expression analyses

The expression analyses of unigenes at the four developmental stages were executed referring to Shamir and Huang et al.’s method [42, 48].

### Gene ontology and KEGG Orthology enrichment analyses of the DEGs

GO and KEGG enrichment analyses of the DEGs were executed referring to Li et al.’s method [8]. The hypergeometric test was used to calculate the enriched *p*-values and the Bonferroni correction method was used for adjusting the p-values [49].

### Quantitative real-time PCR analysis

The remaining RNA samples used in the RNA-seq experiment were used for the synthesis of first-strand cDNA according to Li et al.’s method [8]. Gene expression analysis was executed by real-time PCR as previously described [50]. The housekeeping genes *EF1* and *UBCE2* were used as endogenous references for normalization [51]. Specific primers were designed by primer 3.0 (Addition file 10). The amplification specificity of all target and reference gene was confirmed by observing a single dissociation curve for each pair of primers. The data of similar amplification efficiencies between the target genes and reference genes were used for subsequent analyses. The ΔCt method was used to calculate the values of the target gene relative to the reference gene [52]. Data are calculated as the mean ± standard deviation (SD) of three replications performed in 96-well plates. The data were analysed using CFX Manager™ v3.0.

## Supplementary Information

**Additional file 1 Fig. S1.** Species distribution of top BLAST hits of safflower sequences with other plant species. **Fig. S2.** Eukaryotic of orthologous groups (KOG) classification of assembled unigenes. **Fig. S3** Gene ontology categories of unigenes with significant transcriptional changes during different stages of seed development. **Fig. S4.** The heat map analysis of genes involved in fatty acid biosynthesis among different safflower seed developmental stages. **Fig. S5.** The analysis of the upstream regulatory sequence of *CtFAD2–1.* **Fig. S6.** Nucleotide sequence and cis-acting element of the *CtFAD2–1* gene promoter in safflower. **Table S1.** KEGG categories of nonredundant unigenes in safflower (DOCX 57 kb). **Table S2.** Differentially expressed genes statistical table (DOCX 24 kb). **Table S3.** KEGG orthology enrichment analysis of unigenes with significant transcriptional changes during different stages of seed development (DOCX 29 kb). **Table S4.** KEGG orthology enrichment analysis of unigenes with transcriptional changes involved in seed oil biosynthesis during different stages of seed development.

**Additional file 2 Table S5.** Enzymes/proteins related to lipid accumulation in safflower seeds

## Data Availability

Illumina read data used for expression profiling of the Safflower reference genes have been submitted to the NCBI Sequence Read Archive (SRA) under the accession number SRP186527. All other data supporting our findings can be found in Additional files 1 and 2.

## Abbreviations

DAF: days after flowering
SAD: stearoyl-[acyl-carrier-protein] 9-desaturase
TCM: traditional Chinese medicine
PUFAs: polyunsaturated fatty acids
GC-MS: gas chromatography-mass spectrometry
NCBI: National Center for Biotechnology Information
ORFs: open reading frames
NR: non-redundant protein sequences
KOG: Eukaryotic Orthologue Groups
KEGG: Kyoto Encyclopedia of Genes and Genomes
GO: Gene Ontology
DEGs: differentially expressed genes
LOX: lipoxygenase
ACAA1: acetyl-CoA acyltransferase
JMT: jasmonate O-methyl-transferase
AOX: Allene oxide synthase
MeJA: methyl jasmonate
ACCase: acetyl-CoA carboxylase
FAS: fatty acid synthase
ER: endoplasmic reticulum
LACS: long-chain acyl-CoA synthetases
TAG: Triacylglycerols
DGAT: diacylglyceroltransacylase
PDAT: phospholipid:diacylgly cerolacyltransferase
FAD: fatty acid desaturase
C16:0: palmitic acid
C18:0: stearic acid
C18:1: oleic acid
C18:2: linoleic acid
C18:3: linolenic acid
DHLAT: dihydrolipoamide acetyltransferase
α-PDHC: pyruvate dehydrogenase alpha subunit
β-PDHC: pyruvate dehydrogenase beta subunit
PDP: pyruvate dehydrogenase phosphatase
α-CT: carboxyl transferase alpha subunit
β-CT: carboxyl transferase beta subunit
BC: biotin carboxylase
BCCP: biotin carboxyl carrier protein
MCAAT: malonyl-CoA ACP transacylase
ACP: acyl carrier protein
KAS I, II, III: ketoacyl-ACP synthase I, II, III
KAR: ketoacyl-ACP reductase
HAD: hydroxyacyl-ACP dehydrase
EAR: enoyl-ACP reductase
SAD: stearoyl-ACP desaturase
FAD6: oleate desaturase (chloroplast)
FAD7/8: linoleate desaturase (chloroplast)
FAD2: oleate desaturase
FAD3: linoleate desaturase
FATA/B: acyl-ACP thioesterase A/B
GPAT: glycerol-3-phosphate acyltransferase
LPAAT: 1-acylglycerol-3-phosphate acyltransferase
PAP: phosphatidic acid phosphatase
DGAT1/2 acyl-CoA: diacylglycerolacyltransferase 1/2
PLC: phospholipase C
CK: choline kinase
CCT: choline-phosphate cytidylyltransferase
LPCAT: lysophosphatidylcholine acyltransferase/lyso-PAF acetyltransferase
PDAT: phospholipid:diacylglycerolacyltransferase
DAG-CPT: diacylglycerol cholinephosphotransferase
STC: series test of cluster
HSE: heat-stress response
LTR: low-temperature response
TFs: transcription factors
qRT-PCR: quantitative RT-PCR
