# Supplementary material for: Temporal transcriptome profiling of developing seeds reveals candidate genes involved in oil accumulation in safflower (Carthamus tinctorius L.)
Source: BMC Plant Biol. 2021 Apr 15;21:181. doi: 10.1186/s12870-021-02964-0 (PMC8051040; doi:10.1186/s12870-021-02964-0)
Supplement: Supplementary file 1 — Additional file 1 Fig. S1. Species distribution of top BLAST hits of safflower sequences with other plant species. Fig. S2. Eukaryotic of orthologous groups (KOG) classification of assembled unigenes. Fig. S3 Gene ontology categories of unigenes with significant transcriptional changes during different stages of seed development. Fig. S4. The heat map analysis of genes involved in fatty acid biosynthesis among different safflower seed developmental stages. Fig. S5. The analysis of the upstream regulatory sequence of CtFAD2–1. Fig. S6. Nucleotide sequence and cis-acting element of the CtFAD2–1 gene promoter in safflower. Table S1. KEGG categories of nonredundant unigenes in safflower (DOCX 57 kb). Table S2. Differentially expressed genes statistical table (DOCX 24 kb). Table S3. KEGG orthology enrichment analysis of unigenes with significant transcriptional changes during different stages of seed development (DOCX 29 kb). Table S4. KEGG orthology enrichment analysis of unigenes with transcriptional changes involved in seed oil biosynthesis during different stages of seed development. [file 12870_2021_2964_MOESM1_ESM.docx]

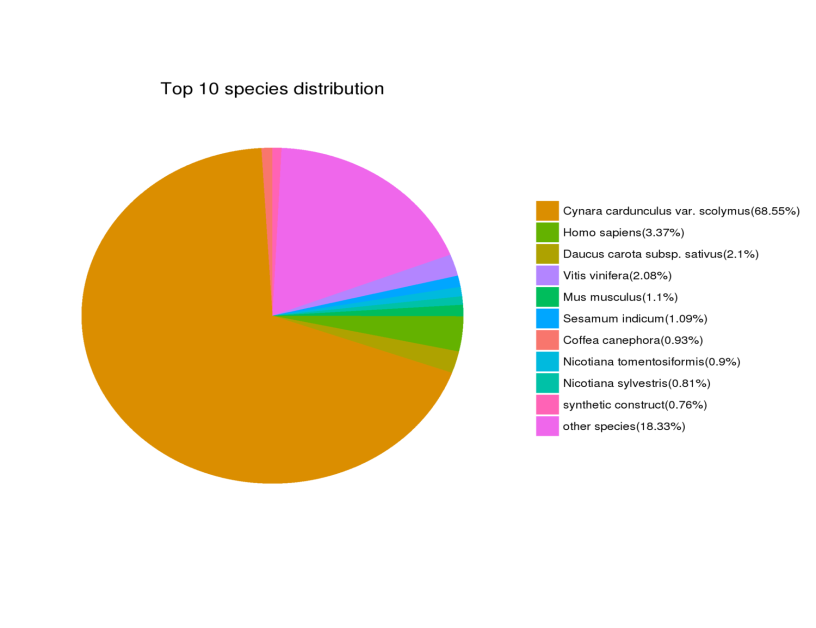


**Figure S1.** Species distribution of top BLAST hits of safflower sequences with other plant species.


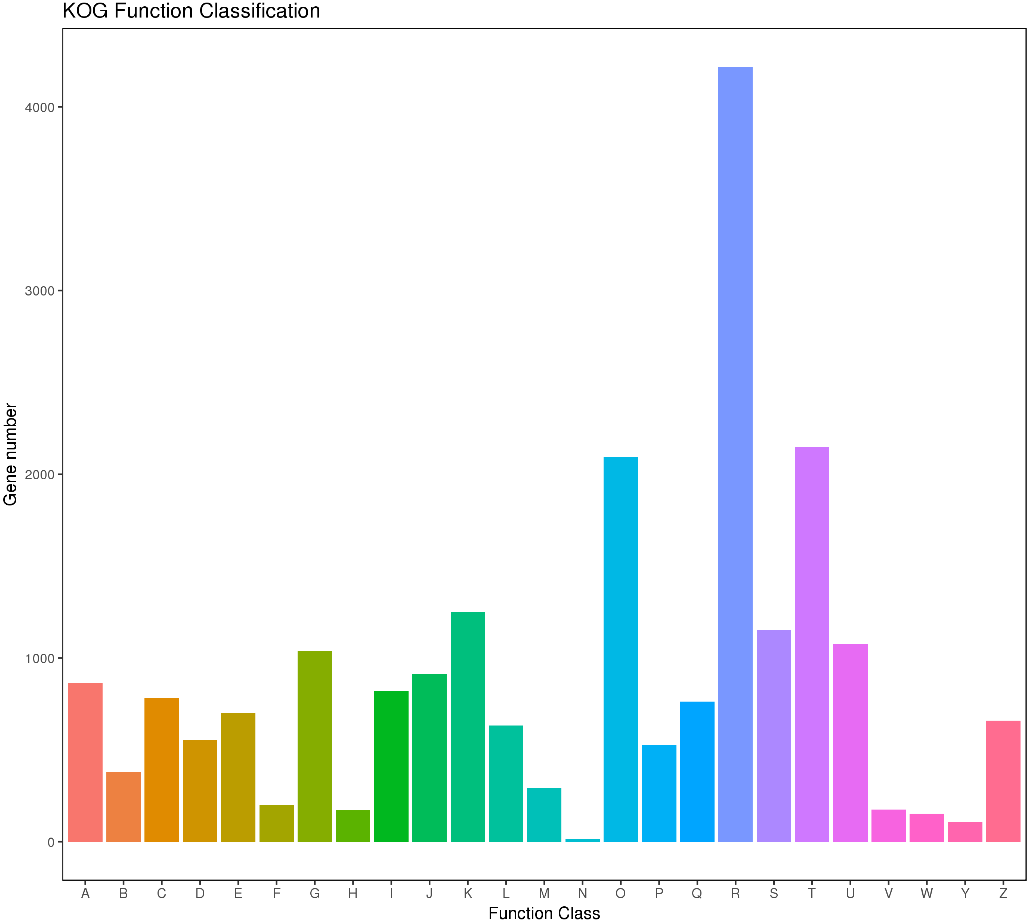


**Figure S2.** Eukaryotic of orthologous groups (KOG) classification of assembled unigenes. A: RNA processing and modification. B: Chromatin structure and dynamics. C: Energy production and conversion. D: Cell cycle control, cell division, chromosome partitioning. E: Amino acid transport and metabolism. F: Nucleotide transport and metabolism. G: Carbohydrate transport and metabolism. H: Coenzyme transport and metabolism. I: Lipid transport and metabolism. J: Translation, ribosomal structure and biogenesis. K: Transcription. L: Replication, recombination and repair. M: Cell wall/membrane/envelope biogenesis. N: Cell motility. O: Posttranslational modification, protein turnover and chaperones. P: Inorganic ion transport and metabolism. Q: Secondary metabolites biosynthesis, transport and catabolism. R: General function prediction only. S: Function unknown T: Signal transduction mechanisms. U: Intracellular trafficking, secretion, and vesicular transport. V: Defense mechanisms. W: Extracellular structures. Y: Nuclear structure. Z: Cytoskeleton.

**10 DAF/14 DAF**


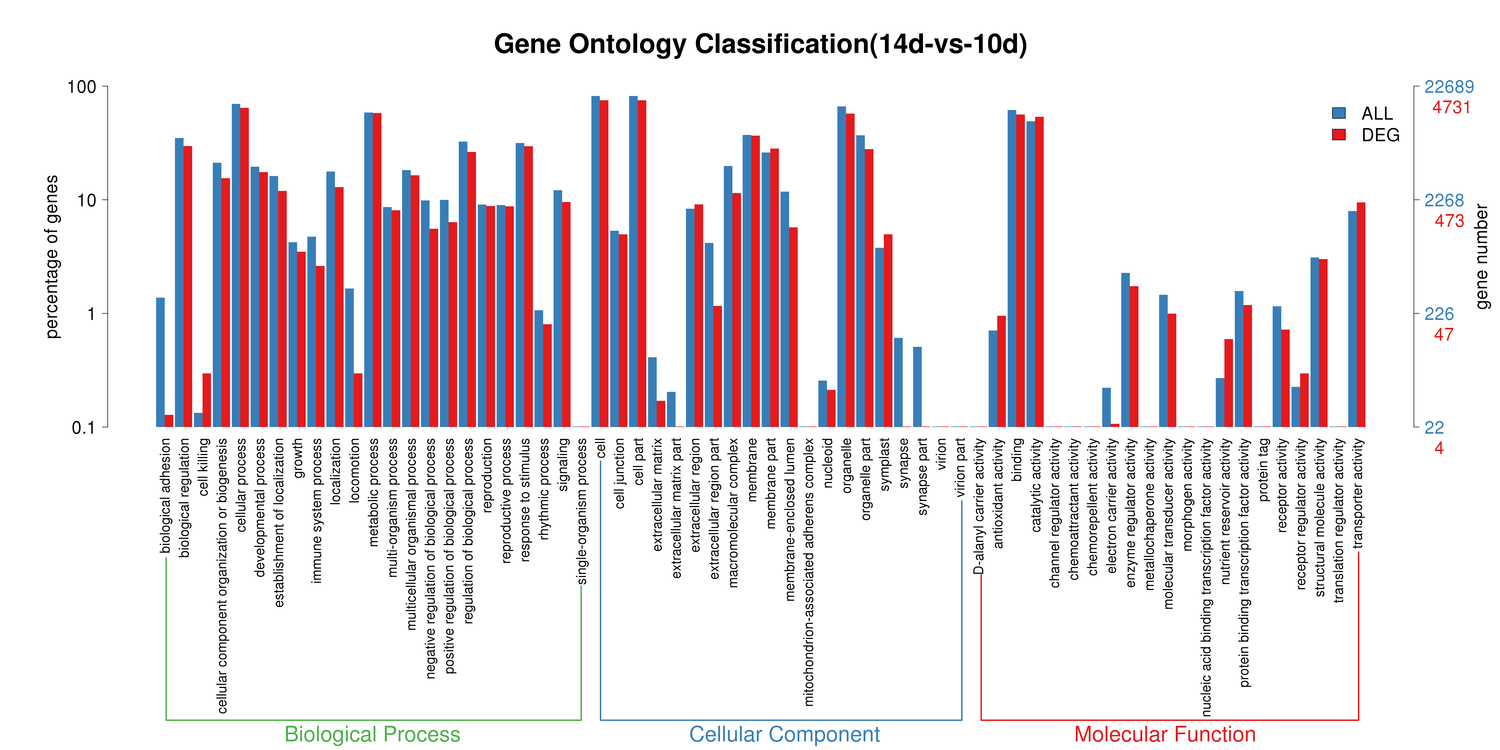


**14 DAF/18 DAF**


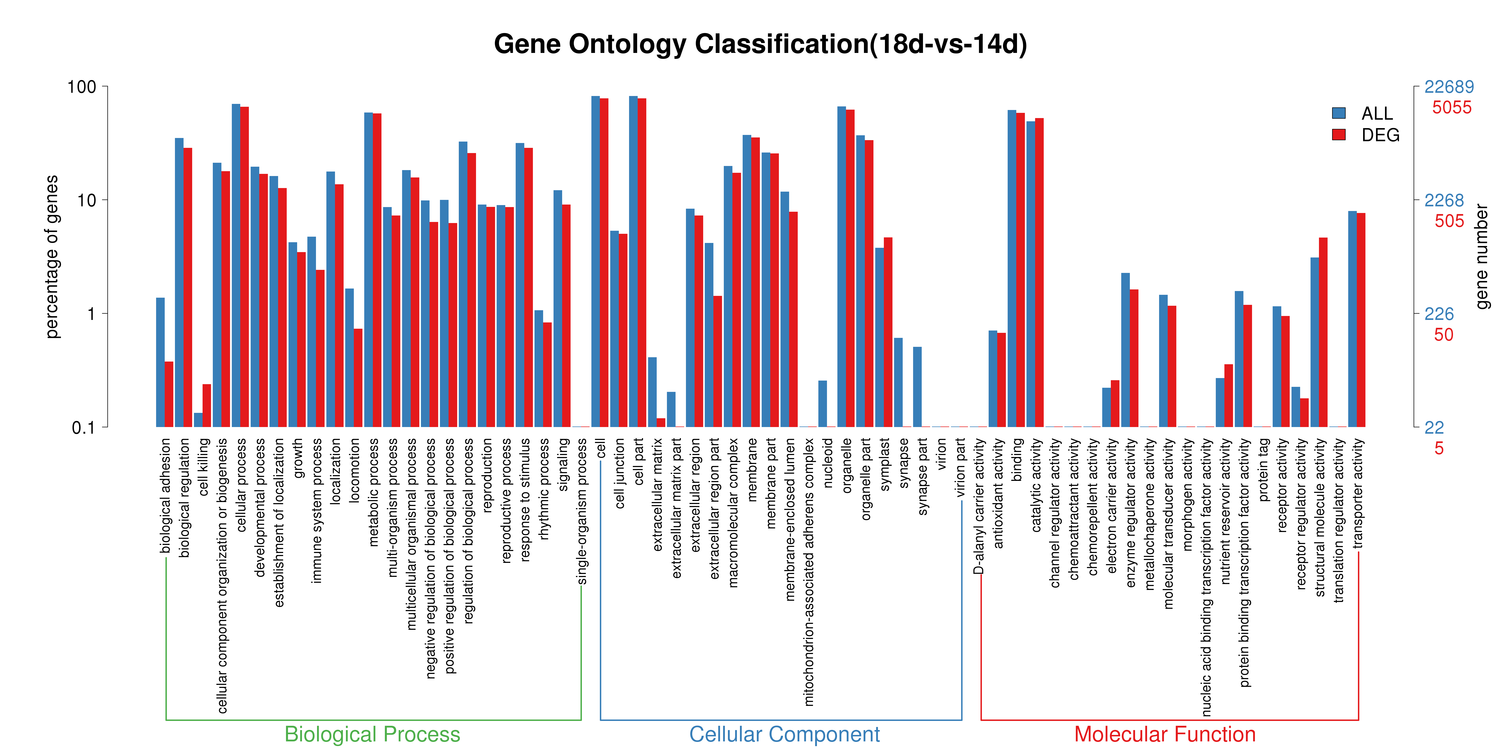


**18 DAF/22 DAF**


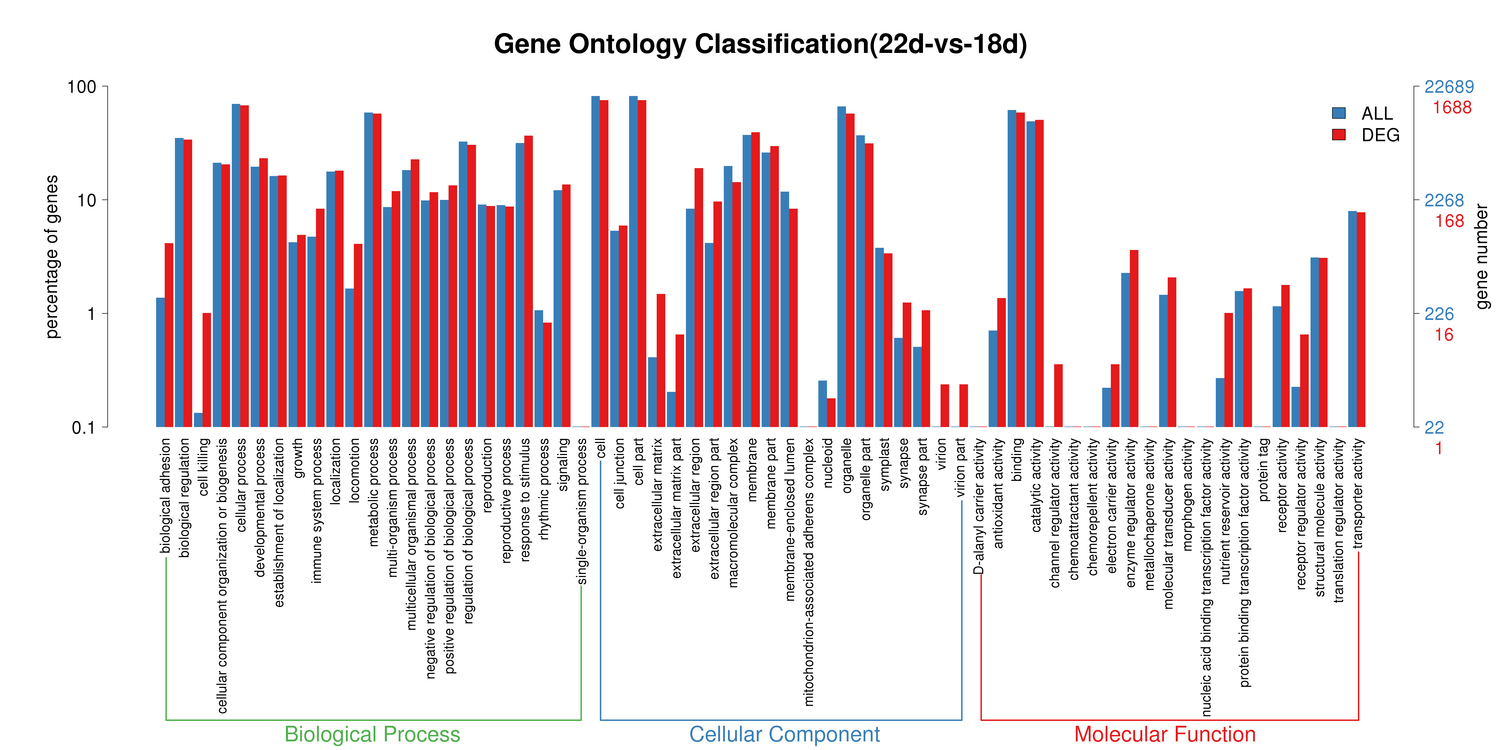


**Figure S3.** Gene Ontology categories of unigenes with significant transcriptional changes during different stages of seed development.

**Figure S4.** The heat map analysis of genes involved in fatty acid biosynthesis among different safflower seed developmental stages.


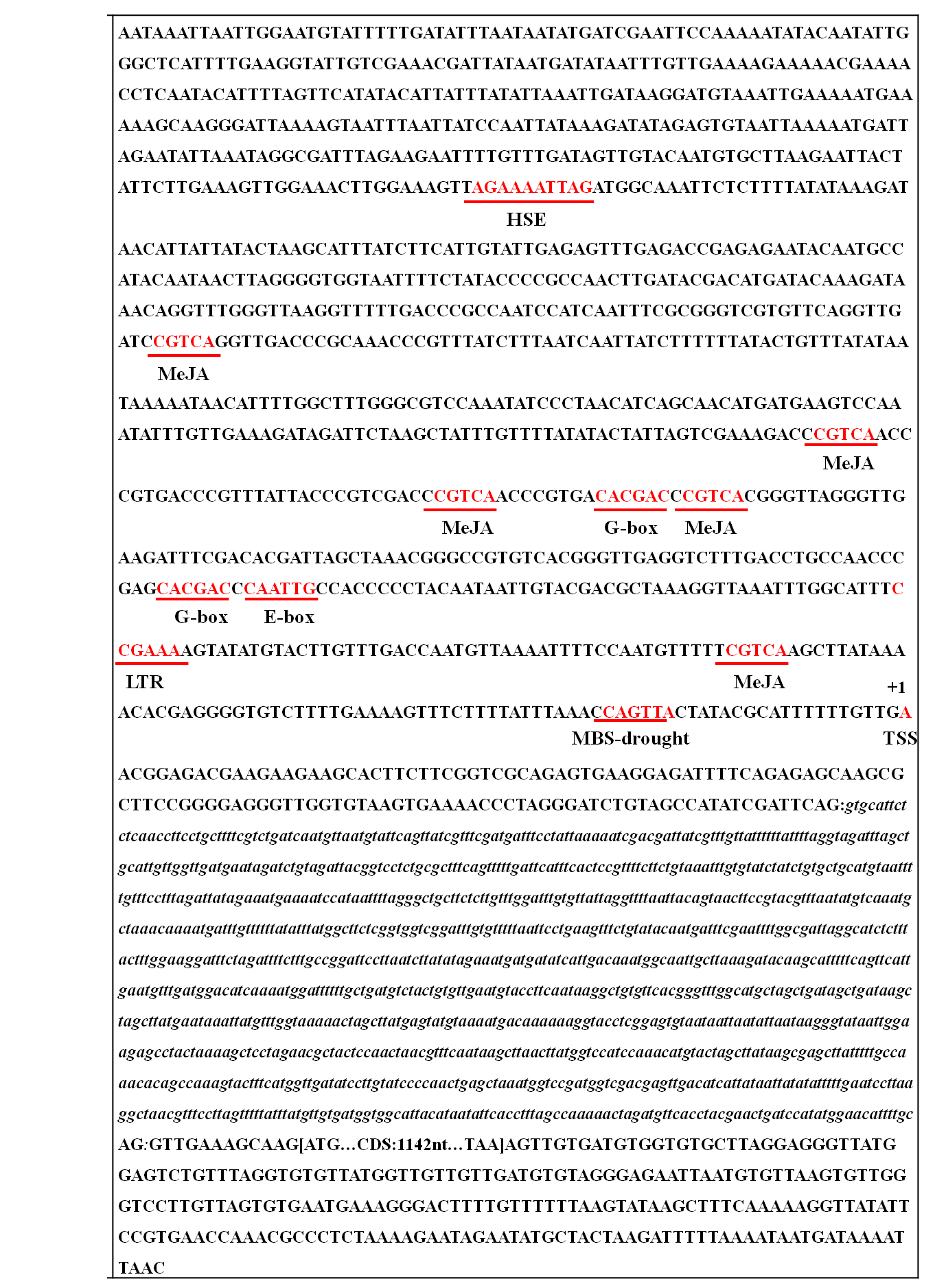


**Figure S5. The analysis of upstream regulatory sequence of *CtFAD2-1***

Note: HSE: Heat-stress response, MeJA: Jasmonic acid methyl ester，G-box: Light response, LTR: Low temperature response, MBS: Drought response, TSS: Transcription initiation site. Lowercase represent intron sequence.


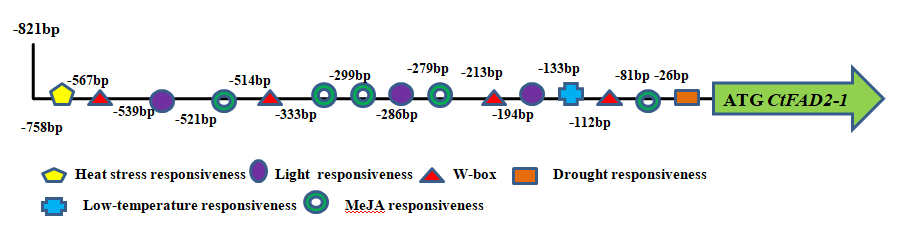


**Figure S6 Nucleotide sequence and cis-acting element of *CtFAD2-1* gene promoter in safflower**

Note: Different colors and shapes represented different cis-acting elements.

**Table S1 KEGG categories of nonredundant unigenes in safflower**

|  | **Pathway** | **Count** | **Pathway ID** |
| --- | --- | --- | --- |
| **1** | **Ribosome** | **403** | **ko03010** |
| **2** | **Carbon metabolism** | **367** | **ko01200** |
| **3** | **Protein processing in endoplasmic reticulum** | **356** | **ko04141** |
| **4** | **Biosynthesis of amino acids** | **331** | **ko01230** |
| **5** | **Spliceosome** | **331** | **ko03040** |
| **6** | **Plant hormone signal transduction** | **323** | **ko04075** |
| **7** | **Endocytosis** | **316** | **ko04144** |
| **8** | **RNA transport** | **301** | **ko03013** |
| **9** | **Starch and sucrose metabolism** | **293** | **ko00500** |
| **10** | **PI3K-Akt signaling pathway** | **270** | **ko04151** |
| **11** | **Cell cycle** | **239** | **ko04110** |
| **12** | **Oxidative phosphorylation** | **234** | **ko00190** |
| **13** | **mRNA surveillance pathway** | **232** | **ko03015** |
| **14** | **Purine metabolism** | **226** | **ko00230** |
| **15** | **Ubiquitin mediated proteolysis** | **207** | **ko04120** |
| **16** | **Glycolysis / Gluconeogenesis** | **205** | **ko00010** |
| **17** | **Focal adhesion** | **200** | **ko04510** |
| **18** | **Cell cycle - yeast** | **196** | **ko04111** |
| **19** | **RNA degradation** | **191** | **ko03018** |
| **20** | **AMPK signaling pathway** | **185** | **ko04152** |
| **21** | **Oocyte meiosis** | **181** | **ko04114** |
| **22** | **Amino sugar and nucleotide sugar metabolism** | **179** | **ko00520** |
| **23** | **Phagosome** | **179** | **ko04145** |
| **24** | **Pyrimidine metabolism** | **172** | **ko00240** |
| **25** | **Regulation of actin cytoskeleton** | **171** | **ko04810** |
| **26** | **Meiosis - yeast** | **168** | **ko04113** |
| **27** | **Sphingolipid signaling pathway** | **154** | **ko04071** |
| **28** | **Ribosome biogenesis in eukaryotes** | **150** | **ko03008** |
| **29** | **Glycerophospholipid metabolism** | **145** | **ko00564** |
| **30** | **Cysteine and methionine metabolism** | **140** | **ko00270** |
| **31** | **cAMP signaling pathway** | **134** | **ko04024** |
| **32** | **Phenylpropanoid biosynthesis** | **132** | **ko00940** |
| **33** | **Lysosome** | **132** | **ko04142** |
| **34** | **Peroxisome** | **129** | **ko04146** |
| **35** | **Phosphatidylinositol signaling system** | **128** | **ko04070** |
| **36** | **Ras signaling pathway** | **125** | **ko04014** |
| **37** | **MAPK signaling pathway** | **123** | **ko04010** |
| **38** | **Pyruvate metabolism** | **122** | **ko00620** |
| **39** | **Wnt signaling pathway** | **121** | **ko04310** |
| **40** | **FoxO signaling pathway** | **117** | **ko04068** |
| **41** | **Fatty acid metabolism** | **116** | **ko01212** |
| **42** | **Nucleotide excision repair** | **113** | **ko03420** |
| **43** | **HIF-1 signaling pathway** | **113** | **ko04066** |
| **44** | **cGMP-PKG signaling pathway** | **112** | **ko04022** |
| **45** | **Glutathione metabolism** | **111** | **ko00480** |
| **46** | **Tight junction** | **111** | **ko04530** |
| **47** | **Carbon fixation in photosynthetic organisms** | **108** | **ko00710** |
| **48** | **Hippo signaling pathway** | **108** | **ko04390** |
| **49** | **Fanconi anemia pathway** | **104** | **ko03460** |
| **50** | **Inositol phosphate metabolism** | **103** | **ko00562** |
| **51** | **DNA replication** | **103** | **ko03030** |
| **52** | **mTOR signaling pathway** | **99** | **ko04150** |
| **53** | **Apoptosis** | **99** | **ko04210** |
| **54** | **Phospholipase D signaling pathway** | **98** | **ko04072** |
| **55** | **Pentose phosphate pathway** | **97** | **ko00030** |
| **56** | **Adherens junction** | **96** | **ko04520** |
| **57** | **Glycine, serine and threonine metabolism** | **94** | **ko00260** |
| **58** | **Pentose and glucuronate interconversions** | **92** | **ko00040** |
| **59** | **Galactose metabolism** | **92** | **ko00052** |
| **60** | **Glycerolipid metabolism** | **92** | **ko00561** |
| **61** | **Photosynthesis** | **91** | **ko00195** |
| **62** | **Glyoxylate and dicarboxylate metabolism** | **91** | **ko00630** |
| **63** | **Rap1 signaling pathway** | **91** | **ko04015** |
| **64** | **Fructose and mannose metabolism** | **88** | **ko00051** |
| **65** | **Calcium signaling pathway** | **88** | **ko04020** |
| **66** | **Arginine and proline metabolism** | **87** | **ko00330** |
| **67** | **MAPK signaling pathway - fly** | **86** | **ko04013** |
| **68** | **Mismatch repair** | **85** | **ko03430** |
| **69** | **Methane metabolism** | **83** | **ko00680** |
| **70** | **NF-kappa B signaling pathway** | **80** | **ko04064** |
| **71** | **Aminoacyl-tRNA biosynthesis** | **78** | **ko00970** |
| **72** | **Protein export** | **76** | **ko03060** |
| **73** | **Homologous recombination** | **76** | **ko03440** |
| **74** | **Valine, leucine and isoleucine degradation** | **74** | **ko00280** |
| **75** | **Proteasome** | **73** | **ko03050** |
| **76** | **Base excision repair** | **73** | **ko03410** |
| **77** | **p53 signaling pathway** | **73** | **ko04115** |
| **78** | **Citrate cycle (TCA cycle)** | **72** | **ko00020** |
| **79** | **Quorum sensing** | **72** | **ko02024** |
| **80** | **N-Glycan biosynthesis** | **71** | **ko00510** |
| **81** | **Porphyrin and chlorophyll metabolism** | **71** | **ko00860** |
| **82** | **Regulation of mitophagy - yeast** | **70** | **ko04139** |
| **83** | **Alanine, aspartate and glutamate metabolism** | **69** | **ko00250** |
| **84** | **Apoptosis - fly** | **68** | **ko04214** |
| **85** | **Hippo signaling pathway - fly** | **68** | **ko04391** |
| **86** | **Fatty acid degradation** | **67** | **ko00071** |
| **87** | **Terpenoid backbone biosynthesis** | **67** | **ko00900** |
| **88** | **ABC transporters** | **67** | **ko02010** |
| **89** | **TGF-beta signaling pathway** | **67** | **ko04350** |
| **90** | **alpha-Linolenic acid metabolism** | **66** | **ko00592** |
| **91** | **2-Oxocarboxylic acid metabolism** | **64** | **ko01210** |
| **92** | **ECM-receptor interaction** | **63** | **ko04512** |
| **93** | **Basal transcription factors** | **61** | **ko03022** |
| **94** | **Phenylalanine, tyrosine and tryptophan biosynthesis** | **60** | **ko00400** |
| **95** | **MAPK signaling pathway - yeast** | **60** | **ko04011** |
| **96** | **Ascorbate and aldarate metabolism** | **59** | **ko00053** |
| **97** | **beta-Alanine metabolism** | **59** | **ko00410** |
| **98** | **Regulation of autophagy** | **57** | **ko04140** |
| **99** | **Metabolism of xenobiotics by cytochrome P450** | **56** | **ko00980** |
| **100** | **SNARE interactions in vesicular transport** | **56** | **ko04130** |
| **101** | **Various types of N-glycan biosynthesis** | **55** | **ko00513** |
| **102** | **Biosynthesis of unsaturated fatty acids** | **55** | **ko01040** |
| **103** | **Tyrosine metabolism** | **54** | **ko00350** |
| **104** | **Ether lipid metabolism** | **54** | **ko00565** |
| **105** | **Drug metabolism - cytochrome P450** | **54** | **ko00982** |
| **106** | **Lysine degradation** | **53** | **ko00310** |
| **107** | **Fatty acid elongation** | **52** | **ko00062** |
| **108** | **RNA polymerase** | **52** | **ko03020** |
| **109** | **VEGF signaling pathway** | **52** | **ko04370** |
| **110** | **Fatty acid biosynthesis** | **51** | **ko00061** |
| **111** | **Cyanoamino acid metabolism** | **51** | **ko00460** |
| **112** | **Sphingolipid metabolism** | **51** | **ko00600** |
| **113** | **Hedgehog signaling pathway - fly** | **48** | **ko04341** |
| **114** | **Ubiquinone and other terpenoid-quinone biosynthesis** | **47** | **ko00130** |
| **115** | **Tryptophan metabolism** | **47** | **ko00380** |
| **116** | **Carotenoid biosynthesis** | **47** | **ko00906** |
| **117** | **Propanoate metabolism** | **46** | **ko00640** |
| **118** | **TNF signaling pathway** | **45** | **ko04668** |
| **119** | **Steroid biosynthesis** | **44** | **ko00100** |
| **120** | **Glycosylphosphatidylinositol(GPI)-anchor biosynthesis** | **44** | **ko00563** |
| **121** | **Jak-STAT signaling pathway** | **43** | **ko04630** |
| **122** | **Phenylalanine metabolism** | **42** | **ko00360** |
| **123** | **Cell adhesion molecules (CAMs)** | **41** | **ko04514** |
| **124** | **Gap junction** | **41** | **ko04540** |
| **125** | **Carbon fixation pathways in prokaryotes** | **40** | **ko00720** |
| **126** | **Sulfur metabolism** | **40** | **ko00920** |
| **127** | **Arginine biosynthesis** | **39** | **ko00220** |
| **128** | **Nitrogen metabolism** | **38** | **ko00910** |
| **129** | **Notch signaling pathway** | **37** | **ko04330** |
| **130** | **Histidine metabolism** | **35** | **ko00340** |
| **131** | **Other glycan degradation** | **35** | **ko00511** |
| **132** | **Hedgehog signaling pathway** | **35** | **ko04340** |
| **133** | **Signaling pathways regulating pluripotency of stem cells** | **35** | **ko04550** |
| **134** | **Flavonoid biosynthesis** | **34** | **ko00941** |
| **135** | **ErbB signaling pathway** | **33** | **ko04012** |
| **136** | **One carbon pool by folate** | **32** | **ko00670** |
| **137** | **Arachidonic acid metabolism** | **31** | **ko00590** |
| **138** | **Photosynthesis - antenna proteins** | **30** | **ko00196** |
| **139** | **Pantothenate and CoA biosynthesis** | **30** | **ko00770** |
| **140** | **Sesquiterpenoid and triterpenoid biosynthesis** | **30** | **ko00909** |
| **141** | **Drug metabolism - other enzymes** | **30** | **ko00983** |
| **142** | **Selenocompound metabolism** | **29** | **ko00450** |
| **143** | **Linoleic acid metabolism** | **29** | **ko00591** |
| **144** | **Tropane, piperidine and pyridine alkaloid biosynthesis** | **29** | **ko00960** |
| **145** | **Folate biosynthesis** | **28** | **ko00790** |
| **146** | **Two-component system** | **28** | **ko02020** |
| **147** | **Cutin, suberine and wax biosynthesis** | **27** | **ko00073** |
| **148** | **Nicotinate and nicotinamide metabolism** | **27** | **ko00760** |
| **149** | **Isoquinoline alkaloid biosynthesis** | **27** | **ko00950** |
| **150** | **Cytokine-cytokine receptor interaction** | **27** | **ko04060** |
| **151** | **Chloroalkane and chloroalkene degradation** | **26** | **ko00625** |
| **152** | **Butanoate metabolism** | **26** | **ko00650** |
| **153** | **Zeatin biosynthesis** | **26** | **ko00908** |
| **154** | **Glycosaminoglycan degradation** | **25** | **ko00531** |
| **155** | **Cell cycle - Caulobacter** | **25** | **ko04112** |
| **156** | **Streptomycin biosynthesis** | **23** | **ko00521** |
| **157** | **Biotin metabolism** | **23** | **ko00780** |
| **158** | **Valine, leucine and isoleucine biosynthesis** | **22** | **ko00290** |
| **159** | **Stilbenoid, diarylheptanoid and gingerol biosynthesis** | **22** | **ko00945** |
| **160** | **Non-homologous end-joining** | **21** | **ko03450** |
| **161** | **Diterpenoid biosynthesis** | **20** | **ko00904** |
| **162** | **Retinol metabolism** | **19** | **ko00830** |
| **163** | **Limonene and pinene degradation** | **19** | **ko00903** |
| **164** | **Bacterial secretion system** | **19** | **ko03070** |
| **165** | **Lysine biosynthesis** | **17** | **ko00300** |
| **166** | **Thiamine metabolism** | **17** | **ko00730** |
| **167** | **Taurine and hypotaurine metabolism** | **16** | **ko00430** |
| **168** | **Vitamin B6 metabolism** | **16** | **ko00750** |
| **169** | **Monobactam biosynthesis** | **15** | **ko00261** |
| **170** | **Sulfur relay system** | **15** | **ko04122** |
| **171** | **Hippo signaling pathway -multiple species** | **15** | **ko04392** |
| **172** | **Steroid hormone biosynthesis** | **13** | **ko00140** |
| **173** | **Neomycin, kanamycin and gentamicin biosynthesis** | **13** | **ko00524** |
| **174** | **Brassinosteroid biosynthesis** | **12** | **ko00905** |
| **175** | **Degradation of aromatic compounds** | **12** | **ko01220** |
| **176** | **Lipopolysaccharide biosynthesis** | **11** | **ko00540** |
| **177** | **Styrene degradation** | **11** | **ko00643** |
| **178** | **Riboflavin metabolism** | **11** | **ko00740** |
| **179** | **Glycosphingolipid biosynthesis - globo series** | **10** | **ko00603** |
| **180** | **C5-Branched dibasic acid metabolism** | **10** | **ko00660** |
| **181** | **Monoterpenoid biosynthesis** | **10** | **ko00902** |
| **182** | **Glycosphingolipid biosynthesis - ganglio series** | **9** | **ko00604** |
| **183** | **Synthesis and degradation of ketone bodies** | **8** | **ko00072** |
| **184** | **Phosphonate and phosphinate metabolism** | **8** | **ko00440** |
| **185** | **Other types of O-glycan biosynthesis** | **8** | **ko00514** |
| **186** | **Naphthalene degradation** | **8** | **ko00626** |
| **187** | **Biosynthesis of ansamycins** | **8** | **ko01051** |
| **188** | **Apoptosis - multiple species** | **8** | **ko04215** |
| **189** | **Tetracycline biosynthesis** | **7** | **ko00253** |
| **190** | **Neuroactive ligand-receptor interaction** | **7** | **ko04080** |
| **191** | **Novobiocin biosynthesis** | **6** | **ko00401** |
| **192** | **Aminobenzoate degradation** | **6** | **ko00627** |
| **193** | **Lipoic acid metabolism** | **5** | **ko00785** |
| **194** | **Mucin type O-Glycan biosynthesis** | **4** | **ko00512** |
| **195** | **Aflatoxin biosynthesis** | **3** | **ko00254** |
| **196** | **Geraniol degradation** | **3** | **ko00281** |
| **197** | **Benzoate degradation** | **3** | **ko00362** |
| **198** | **Glycosaminoglycan biosynthesis - chondroitin sulfate / dermatan sulfate** | **3** | **ko00532** |
| **199** | **Atrazine degradation** | **3** | **ko00791** |
| **200** | **Flavone and flavonol biosynthesis** | **3** | **ko00944** |
| **201** | **Caffeine metabolism** | **2** | **ko00232** |
| **202** | **Chlorocyclohexane and chlorobenzene degradation** | **2** | **ko00361** |
| **203** | **Bisphenol degradation** | **2** | **ko00363** |
| **204** | **Fluorobenzoate degradation** | **2** | **ko00364** |
| **205** | **D-Glutamine and D-glutamate metabolism** | **2** | **ko00471** |
| **206** | **Polyketide sugar unit biosynthesis** | **2** | **ko00523** |
| **207** | **Toluene degradation** | **2** | **ko00623** |
| **208** | **Glucosinolate biosynthesis** | **2** | **ko00966** |
| **209** | **Glycosaminoglycan biosynthesis - heparan sulfate / heparin** | **1** | **ko00534** |
| **210** | **Peptidoglycan biosynthesis** | **1** | **ko00550** |
| **211** | **Polycyclic aromatic hydrocarbon degradation** | **1** | **ko00624** |
| **212** | **Caprolactam degradation** | **1** | **ko00930** |
| **213** | **Anthocyanin biosynthesis** | **1** | **ko00942** |
| **214** | **Betalain biosynthesis** | **1** | **ko00965** |
| **215** | **Biosynthesis of siderophore group nonribosomal peptides** | **1** | **ko01053** |

**Table S2** **Differentially expressed genes statistical table.**

| **Case** | **Control** | **Up_diff** | **Down_diff** | **Total_diff(pvalue<0.05&\|log2FC\|>1)** |
| --- | --- | --- | --- | --- |
| 22d_1,22d_2,22d_3(22d) | 18d_1,18d_2,18d_3(18d | 2511 | 1716 | 4227 |
| 22d_1,22d_2,22d_3(22d) | 10d_1,10d_2,10d_3(10d) | 9681 | 8484 | 18165 |
| 18d_1,18d_2,18d_3(18d) | 14d_1,14d_2,14d_3(14d) | 5830 | 5064 | 10894 |
| 22d_1,22d_2,22d_3(22d) | 14d_1,14d_2,14d_3(14d) | 7469 | 6160 | 13629 |
| 18d_1,18d_2,18d_3(18d) | 10d_1,10d_2,10d_3(10d) | 8567 | 8164 | 16731 |
| 14d_1,14d_2,14d_3(14d) | 10d_1,10d_2,10d_3(10d) | 5203 | 4577 | 9780 |

Note: 1) Case：experimental group name；2) Control：control group name；3) Up_diff：the numbers of upregulated unigenes with significant difference；4) Down_diff：the numbers of downregulated unigenes with significant difference；5）Total_diff：the total numbers of unigenes with significant difference.

**Table S3. KEGG Orthology enrichment analysis of unigenes with significant transcriptional changes during different stages of seed development.**

**14 DAF/10 DAF**

| KEGG Pathway | DEGs | P_value | Enrichment_score |
| --- | --- | --- | --- |
| Ribosome | 137 | 2.33E-16 | 1.912220844 |
| Plant hormone signal transduction | 106 | 7.49E-12 | 1.845975232 |
| Starch and sucrose metabolism | 95 | 1.89E-10 | 1.823805461 |
| Carbon metabolism | 79 | 0.025086 | 1.210831063 |
| Biosynthesis of amino acids | 74 | 0.012336 | 1.25755287 |

**18 DAF/14 DAF**

| KEGG Pathway | DEGs | P_value | Enrichment_score |
| --- | --- | --- | --- |
| Ribosome | 202 | 2.51E-36 | 2.221606 |
| Carbon metabolism | 105 | 0.002282 | 1.268072 |
| Biosynthesis of amino acids | 96 | 0.002122 | 1.285476 |
| Plant hormone signal transduction | 94 | 0.002079 | 1.28987 |
| Starch and sucrose metabolism | 85 | 0.003522 | 1.285796 |

**22 DAF/18 DAF**

| KEGG Pathway | DEGs | P_value | Enrichment_score |
| --- | --- | --- | --- |
| PI3K-Akt signaling pathway | 39 | 4.20E-05 | 1.869712 |
| Phagosome | 38 | 1.75E-09 | 2.747923 |
| Biosynthesis of amino acids | 32 | 0.076028 | 1.251399 |
| Focal adhesion | 31 | 5.55E-05 | 2.006345 |
| Starch and sucrose metabolism | 30 | 0.044204 | 1.325341 |

**Table S4. KEGG Orthology enrichment analysis of unigenes with transcriptional changes involved in seed oil biosynthesis during different stages of seed development.**

**14 DAF/10 DAF**

| KEGG Pathway | DEGs | P_value | Enrichment_score | |  |
| --- | --- | --- | --- | --- | --- |
| fatty acid biosynthesis | 26 | 1.24E-08 | | 2.867647 | |
| biosynthesis of unsaturated fatty acids | 24 | 1.84E-06 | | 2.454545 | |
| fatty acid elongation | 14 | 0.033301 | | 1.514423 | |
| glycerolipid metabolism | 19 | 0.192001 | | 1.161684 | |
| glycerophospholipid metabolism | 25 | 0.515215 | | 0.854527 | |
| Linoleic acid metabolism | 9 | 0.023359 | | 1.745689 | |

**18 DAF/14 DAF**

| KEGG Pathway | DEGs | P_value | Enrichment_score |
| --- | --- | --- | --- |
| fatty acid biosynthesis | 15 | 0.092890 | 1.303592 |
| biosynthesis of unsaturated fatty acids | 19 | 0.013873 | 1.531128 |
| fatty acid elongation | 13 | 0.271684 | 1.108053 |
| glycerolipid metabolism | 21 | 0.417637 | 1.011701 |
| glycerophospholipid metabolism | 28 | 0.799671 | 0.008550 |
| Linoleic acid metabolism | 12 | 0.006416 | 1.834010 |

**22 DAF/18 DAF**

| KEGG Pathway | DEGs | P_value | Enrichment_score |
| --- | --- | --- | --- |
| fatty acid biosynthesis | 1 | 0.913427 | 0.253807 |
| biosynthesis of unsaturated fatty acids | 12 | 0.000214 | 2.824181 |
| fatty acid elongation | 5 | 0.21058 | 1.244631 |
| glycerolipid metabolism | 7 | 0.417896 | 0.736704 |
| glycerophospholipid metabolism | 8 | 0.798762 | 0.968273 |
| Linoleic acid metabolism | 6 | 0.005514 | 2.7618803 |
